# Disease-related patterns of in vivo pathology in Corticobasal syndrome

**DOI:** 10.1007/s00259-018-4104-2

**Published:** 2018-08-08

**Authors:** Flavia Niccolini, Heather Wilson, Stephanie Hirschbichler, Tayyabah Yousaf, Gennaro Pagano, Alexander Whittington, Silvia P. Caminiti, Roberto Erro, Janice L. Holton, Zane Jaunmuktane, Marcello Esposito, Davide Martino, Ali Abdul, Jan Passchier, Eugenii A. Rabiner, Roger N. Gunn, Kailash P. Bhatia, Marios Politis, for the Alzheimer’s Disease Neuroimaging Initiative

**Affiliations:** 1 Neurodegeneration Imaging Group, Maurice Wohl Clinical Neuroscience Institute, Institute of Psychiatry, Psychology and Neuroscience (IoPPN), King’s College London, 125 Coldharbour Lane, Camberwell, London, SE5 9NU UK; 2 Sobell Department of Motor Neuroscience, UCL Institute of Neurology, London, UK; 3 Division of Brain Sciences, Department of Medicine, Imperial College London, London, UK; 4 Center for Neurodegenerative Diseases (CEMAND) Department of Medicine, Surgery and Dentistry, University of Salerno, Salerno, Italy; 5 Division of Neuropathology, UCL Institute of Neurology, London, UK; 6 Department of Neurosciences, Reproductive Sciences and Odontostomatology, Federico II University of Naples, Naples, Italy; 7 Department of Clinical Neurosciences, Cumming School of Medicine, University of Calgary, Calgary, Canada; 8 Imanova Ltd, Centre for Imaging Sciences, Hammersmith Hospital, London, UK; 9 Centre for Neuroimaging Sciences, Institute of Psychiatry, Psychology and Neuroscience, King s College London, London, UK

**Keywords:** Corticobasal syndrome, Tau, PET, MRI

## Abstract

**Purpose:**

To assess disease-related patterns of in vivo pathology in 11 patients with Corticobasal Syndrome (CBS) compared to 20 healthy controls and 33 mild cognitive impairment (MCI) patients due to Alzheimer’s disease.

**Methods:**

We assessed tau aggregates with [^18^F]AV1451 PET, amyloid-β depositions with [^18^F]AV45 PET, and volumetric microstructural changes with MRI. We validated for [^18^F]AV1451 standardised uptake value ratio (SUVRs) against input functions from arterial metabolites and found that SUVRs and arterial-derived distribution volume ratio (DVRs) provide equally robust measures of [^18^F]AV1451 binding.

**Results:**

CBS patients showed increases in [^18^F]AV1451 SUVRs in parietal (*P* < 0.05) and frontal (*P* < 0.05) cortices in the affected hemisphere compared to healthy controls and in precentral (*P* = 0.008) and postcentral (*P* = 0.034) gyrus in the affected hemisphere compared to MCI patients. Our data were confirmed at the histopathological level in one CBS patient who underwent brain biopsy and showed sparse tau pathology in the parietal cortex co-localizing with increased [^18^F]AV1451 signal. Cortical and subcortical [^18^F]AV45 uptake was within normal levels in CBS patients. In parietal and frontal cortices of the most affected hemisphere we found also grey matter loss (*P* < 0.05), increased mean diffusivity (*P* < 0.05) and decreased fractional anisotropy (*P* < 0.05) in CBS patients compared to healthy controls and MCI patients. Grey matter loss and white matter changes in the precentral gyrus of CBS patients were associated with worse motor symptoms.

**Conclusions:**

Our findings demonstrate disease-related patterns of in vivo tau and microstructural pathology in the absence of amyloid-β, which distinguish CBS from non-affected individuals and MCI patients.

**Electronic supplementary material:**

The online version of this article (10.1007/s00259-018-4104-2) contains supplementary material, which is available to authorized users.

## Introduction

Corticobasal syndrome (CBS) is a rare sporadic neurodegenerative disorder clinically characterised by asymmetric rigidity and apraxia with other features such as cortical sensory loss, alien limb behaviour, conjugate ocular movement abnormalities, bradykinesia, myoclonus and dementia [1]. The core neuropathological feature of corticobasal degeneration is abnormal accumulation of hyperphosphorylated 4-repeat tau (4R) in the form of neurofibrillary tangles, neuropil threads and coiled bodies together with astrocytic plaques [2]. The clinical diagnostic accuracy of CBS is poor due to the overlapping clinical features with other neurodegenerative disorders such as Alzheimer’s disease (AD), progressive supranuclear pPalsy (PSP) and tau-positive forms of frontotemporal dementia (FTD). Only 25–56% of cases are correctly diagnosed *antemortem* [3]. Therefore, disease-related patterns of pathology that could be assessed in vivo with non-invasive procedures such as neuroimaging could aid accurate diagnosis, provide neuropathological insight and help in assessing response of disease-modifying treatments.

Recently, PET with specific radioligands binding to aggregated tau has provided a unique opportunity to assess tau pathology in living humans [4]. Autoradiography studies with *post-mortem* human tissue have shown that [^18^F]AV1451 selectively binds to hyperphosphorylated tau over amyloid-β plaques [5]. [^18^F]AV1451 binds with higher affinity to paired helical filaments of 3R over 4R tau isoforms; however, autoradiography studies in *post-mortem* tissue have shown specific binding in patients with CBS [5–7]. Recently, an in vivo [^18^F]AV1451 PET study has shown increased tau uptake in the motor cortex, corticospinal tract, and basal ganglia in the hemisphere contralateral to the most affected body side of six patients with CBS compared to healthy controls and patients with AD and PSP [8]. Another [^18^F]AV1451 PET study has demonstrated increased tau binding in the putamen, globus pallidus, thalamus and precentral grey and white matter in the hemisphere contralateral to the clinically most affected side in six CBS patients [9]. Previous MRI studies have shown grey matter loss and white matter changes in precentral, superior frontal, and fusiform gyri, putamen and globus pallidus in CBS patients [10, 11].

However, these neuroimaging studies are limited by the small sample size, commonly assessing a handful of CBS patients, the use of a single imaging modality lack of arterial input function for assessing [^18^F]AV1451 binding and the lack of any evidence for confirmation of in vivo findings at the histopathological level. Moreover, there is additional scientific advantage regarding neuroimaging potential by comparing disease-related patterns of in vivo pathology in patients with CBS to early stages of AD such as in patients with mild cognitive impairment (MCI) due to AD.

In this study, by using multimodal PET and MR neuroimaging, we sought to identify disease-related patterns of in vivo pathology of tau aggregates using [^18^F]AV1451 PET, amyloid-β deposition with [^18^F]AV45, grey matter and white matter microstructural changes with 3-T MRI, in a group of patients with CBS compared to age-matched healthy controls and a group of patients with MCI due to AD. Our study also included validation of simplified SUVR analyses in relation to optimised arterial input function kinetic modelling approach for [^18^F]AV1451 data, and histopathological examination of a brain biopsy in one patient with CBS.

## Materials and methods

### Participants

Eleven patients with CBS according to the new criteria for the diagnosis of CBS [3] were recruited from specialist movement disorders clinics at King’s College Hospital NHS Foundation Trust and National Hospital of Neurology and Neurosurgery, Queen Square, London (Table 1). Twenty age- and sex-matched healthy individuals with no history of neurological or psychiatric disorders served as the control group. Fifteen of these healthy controls were selected from the ADNI database. Thirty-three age- and sex-matched patients with MCI due to AD [12] from the ADNI database were also included for comparisons of imaging data with the group of patients with CBS (Table 1).

**Table 1:** Clinical characteristics of patients with corticobasal syndrome, mild cognitive impairment and healthy controls

	HC	CBS patients	MCI patients
No (M, %)	20 (10 M, 50.0%)	11 (5 M, 45.4%)	33 (19 M, 57.6%)
Age in years (mean ± SD)	72.4 (±4.8)	69.2 (±6.8)	75.4 (±5.8)
Disease duration (years ±SD)	–	4.82 (±2.2)	9.6 (±6.7)
MMSE (mean ± SD)	29.67 (±0.8)	23.64 (±5.4)*	27.6 (±3.0)^¥^
MOCA (mean ± SD)	29.33 (±1.6)	17.82 (±6.5)***	23.5 (±4.1)^¥^

CBS=Corticobasal Syndrome; HC = Healthy Controls; MCI = Mild Cognitive Impairment; MMSE = Mini Mental Status Examination; MoCA = Montreal Cognitive Assessment. Mean (±SD) time delay between clinical examination and imaging assessments = 20.7 (±15.5) days. *P < 0.05, ***P < 0.001 between corticobasal syndrome patients and healthy controls. ^**¥**^*P* < 0.01 between CBS patients and MCI patients

All participants screened successfully to undertake PET and MRI scanning under scanning safety criteria (http://www.mrisafety.com; https://www.gov.uk/government/publications/arsac-notes-for-guidance) and had no history of other neurological or psychiatric disorders. Details of clinical assessments can be found in Supplemental Methods. The study was approved by the institutional review boards and the research ethics committee. Written informed consent was obtained from all study participants in accordance with the Declaration of Helsinki.

### Image data analysis

#### PET data analysis

The Molecular Imaging and Kinetic Analysis Toolbox software package (MIAKAT™: www.miakat.org), implemented in MATLAB® (The Mathworks, Natick, MA, USA) was used to carry out image processing and kinetic modelling. MIAKAT™ combines in-house code with wrappers for FMRIB Software Library (FSL, http://fsl.fmrib.ox.ac.uk/fsl/fslwiki/) and Statistical Parametric Mapping (SPM, http://www.fil.ion.ucl.ac.uk/spm/) commands in order to provide state-of-the-art functionality within a coherent analysis framework. Individual PET frames were corrected for head motion using frame-by-frame rigid registration using a frame with high signal-to-noise ratio as reference. The MIAKAT™ processing pipeline was followed, ensuring that all quality control steps were completed.

#### [^18^F]AV1451 arterial input function

All patients with CBS and the healthy controls scanned at Imanova underwent arterial sampling for measurements of radioactivity concentrations. One patient with CBS was unable to tolerate arterial cannulation and, therefore, metabolite analysis was not performed for this patient. [^18^F]AV1451 parent fraction over the course of the PET scan was determined by HPLC using the Hilton column switching method [13]. Plasma input function of unmetabolised radioligand was generated using the continuous and discrete plasma samples. The arterial input function was obtained by plasma-to-whole blood radios fitted with a single exponential fit and a sigmoid fit for parent fraction [14].

#### [^18^F]AV1451 pet

[^18^F]AV1451 total volume of distribution (V_T_) was generated using the two-tissue compartmental model (2-TCM) with blood volume correction [14, 15]. [^18^F]AV1451 V_T_ reflects the equilibrium ratio of [^18^F]AV1451 concentration in the tissue vs plasma [16]. To quantify specific binding of [^18^F]AV1451, indirect distribution volume ratio (DVR) was estimated from compartmental modelling with arterial inputs, calculated as Logan V_T_^tissue^ /V_T_^ref^ with cerebellum grey matter, excluding the dentate nucleus, as reference. [^18^F]AV1451 DVR has been shown to correlate with 2-TCM Logan V_T_ and yields high quality parametric maps for tau quantification with PET [14, 17]; therefore, [^18^F]AV1451 DVR parametric maps were generated from Logan V_T_ [17].

For the clinical application of [^18^F]AV1451 and for comparison with previous studies without arterial inputs, we also quantified [^18^F]AV1451 using standardised uptake value ratio 60–80 (SUVR) min post-injection with cerebellar grey matter excluding the dentate nucleus as the reference tissue [18, 19]. SUV was generated by correcting absolute radioactivity concentrations (C; kBq/mL) for subject body weight (BW; kg) and injected dose (ID; MBq): SUV=C/(ID/BW).

#### [^18^F]AV45 pet

Quantification of [^18^F]AV45 in vivo was expressed as SUVR 50–60 min post-injection. SUVRs were calculated as radioactivity concentration in each region of interest tissue divided by the radioactivity concentration in the cerebellum grey matter as the reference tissue for no amyloid-specific [^18^F]AV45 uptake. In line with previous studies, the cortical to cerebellar SUVRs values reached a plateau within 50 min; therefore, the time window 50–60 min post-injection was taken as a suitable representative sample for analysis [20].

### MRI data analysis

#### FreeSurfer analysis

FreeSurfer image analysis suite was used to derive measures of cortical thickness and deep grey matter nuclei volume. Cortical thickness was measured as the distance from the grey and white matter boundary to the corresponding pial surface. Reconstructed data sets were visually inspected to ensure accuracy of registration, skull stripping, segmentation, and cortical surface reconstruction. Subcortical structure volumes were derived by automated procedures, which automatically assign a neuroanatomical label to each voxel in an MRI volume based on probabilistic information automatically estimated from a manually labelled training set [21]. All individual nuclei volumes were normalised for intracranial volume automatically generated by FreeSurfer [22].

#### DTI analysis

Diffusion data analysis was performed using FSL Diffusion Toolbox (FDT) (FMRIB Centre Software Library, Oxford University). Each phase encoding direction image set, blip-up and blip-down, was corrected for motion and eddy current-related distortions [23]. Diffusion tensors were estimated on a voxel-by-voxel basis using DTIfit within the FMRIB Diffusion Toolbox to obtain mapping of mean diffusivity (MD) and fractional anisotropy (FA). Voxel-wise tract-based spatial statistics (TBSS) [24] was used to analyse FA and MD between healthy controls and patients with CBS and MCI. All subjects’ FA data were registered into a common space and mean FA skeleton was created using a threshold of 0.2. The group differences were calculated using a voxel-by-voxel non-parametric test (500 permutations) and the results reported after threshold-free cluster enhancement to avoid an arbitrary threshold for the initial cluster formation [25]. Results were corrected for multiple comparisons at *P* < 0.05.

Neuropathological analysis can be found in Supplemental methods.

### Statistical analysis

Statistical analysis and graph illustration were performed with SPSS (version 20 Chicago, IL, USA) and GraphPad Prism (version 6.0c) for MAC OS X, respectively. For all variables, variance homogeneity and Gaussianity were tested with Bartlett and Kolmogorov-Smirnov tests. Multivariate analysis of variance (MANOVA) was used to assess groups’ difference in clinical, PET and MR imaging data. If the overall multivariate test was significant, *P*-values for each variable were calculated following Bonferroni’s multiple comparisons test. For analysis of asymmetric [^18^F]AV1451 uptake, contralateral to the clinically most affected side of the body, the most affected hemisphere was flipped to the same side for each subject (most affected left hemisphere = 3 CBS patients; most affected right hemisphere = 8 CBS) to allow comparison of the most and least affected hemisphere in the group of 11 CBS patients. Since inter-scanner variability, reconstruction techniques, and different implementations of scatter and attenuation corrections in PET and MRI images from various sites could have affect our results, we repeated the analysis by co-varying between data acquired at our center and the ADNI dataset. We interrogated correlations between PET and clinical data using Spearman’s *r* correlation coefficient and we applied the Benjamini-Hochberg correction. *P*-values for each variable were calculated following Benjamini-Hochberg multiple-comparisons test in order to reduce false discovery rate. We set the false discovery rate cut-off at 0.05. All data are presented as mean ± SD, and the level *α* was set for all comparisons at *P* < 0.05, Benjamini-Hochberg corrected. For voxel-wise statistics appropriately weighted contrasts were used to derive Z-scores on a voxel basis using the general linear model; threshold for statistical significant was set to *P* < 0.05.

## Results

### Clinical assessments

Patients with CBS had worse cognitive function (MMSE *P* = 0.017; MoCA *P* = 0.007; PSPSR-II mental exam *P* = 0.008) and worse symptoms of frontal lobe dysfunction (FAB: *P* < 0.001) compared to the group of healthy controls (Table S1) and compared to the group of MCI patients (MMSE *P* = 0.003; MoCA *P* = 0.001; Table 1). Three CBS patients were unable to perform the CANTAB® battery due to severe motor and cognitive impairment (Subject 7: MMSE = 16, MoCA = 7, UPDRS-III = 64; Subject 9: MMSE = 17, MoCA = 15, UPDRS-III = 63; Subject 11: MMSE = 18, MoCA = 12, UPDRS-III = 85). CBS patients performed worse than healthy controls in the assessments of psychomotor speed [five choice median reaction time (*P* = 0.011) and median movement time (*P* = 0.017)], attention [rapid visual information processing A-time (*P* = 0.048) and median latency (*P* = 0.009)] and episodic memory [delayed match to sample % correct (*P* = 0.032) and probability of given error (*P* = 0.004); Table S2]. CBS patients had higher burden of neuropsychiatric symptoms as measured by the NPI (*P* = 0.013), GDS (*P* = 0.024) and HDRS (*P* = 0.003). Non-motor symptoms burden was also higher in our group of CBS patients compared to the group of healthy controls (UPDRS-I: *P* = 0.006; ESS: *P* = 0.040; SCOPA-AUT: *P* = 0.008; Table S1).

### [^18^F]AV1451 PET findings

We first validated use of simplified SUVR analyses in relation to optimised arterial input function kinetic modelling approach for [^18^F]AV1451. For 10 CBS patients and five healthy controls, arterial quantification of [^18^F]AV1451 was carried out using the 2-TC model with blood volume correction, to generate regional V_T_ values. The cerebellum grey matter, excluding the dentate nucleus, has been used as a reference region for quantification of [^18^F]AV1451 in simplified model including SUVR analysis. In our data set, there was no difference (*P* > 0.10) in V_T_ cerebellum grey matter between CBS patients (mean ± SD: 5.29 ± 1.1) and healthy controls (mean ± SD: 5.22 ± 1.4). Therefore, cerebellum grey matter is a suitable reference region for simplified analysis methods. We investigated differences in cortical and subcortical [^18^F]AV1451 uptake using Logan DVR (V_T_^tissue^ /V_T_^ref^) and SUVR. No significant differences were found between mean cortical [^18^F]AV1451 SUVRs and [^18^F]AV1451 Logan DVRs in our group of CBS patients (Table S3) and healthy controls (all *P* > 0.10; Table S4; Fig. S1). These results validate the use of SUVR as a reliable, simplified method for the quantification of [^18^F]AV1451. [^18^F]AV1451 SUVR was used to carry out group comparisons and correlations.

**Fig. 1:**
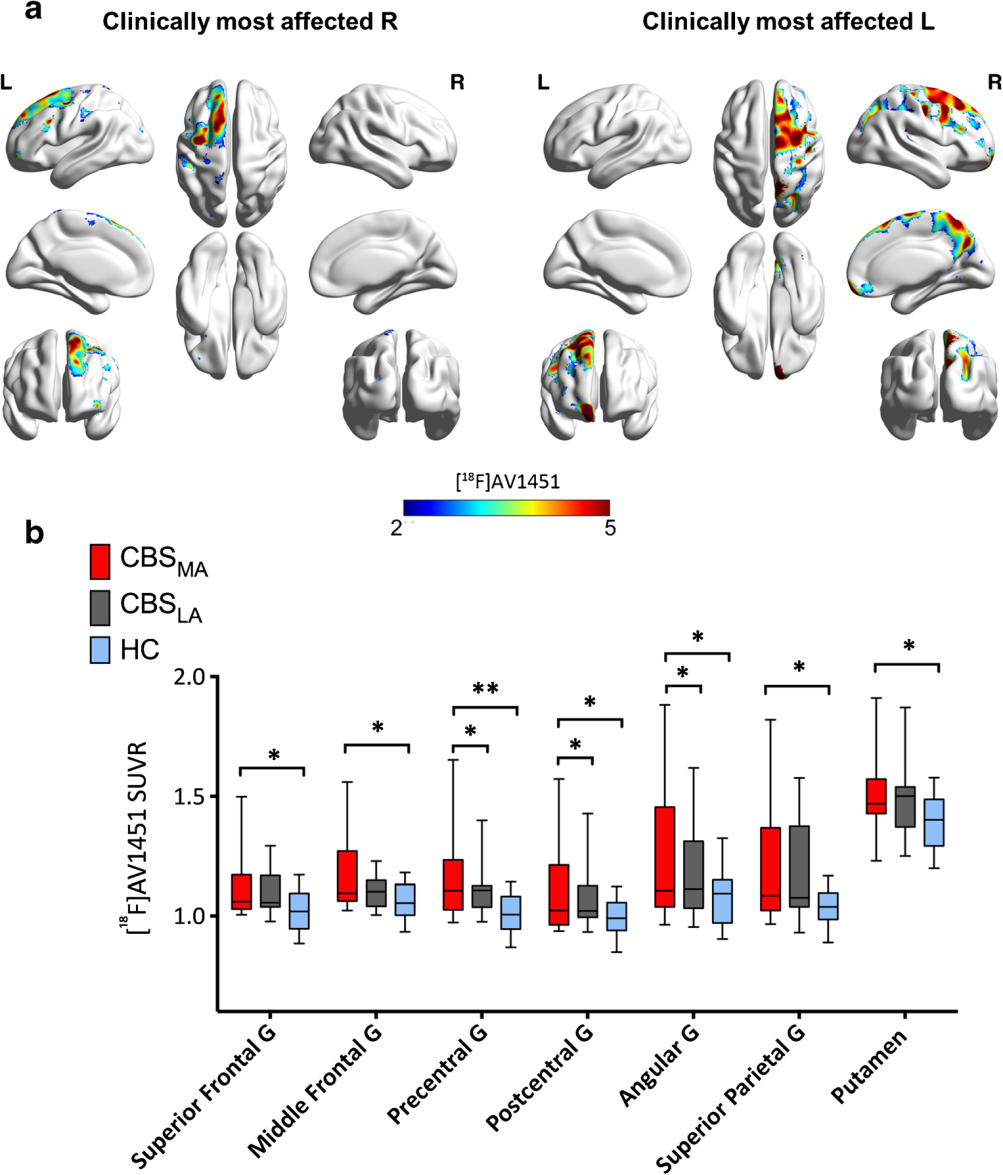
Increased tau deposition in the most and least affected side of corticobasal syndrome patients. (A) Voxel-wise z-score maps for [^18^F]AV1451 standardized uptake value ratios (SUVR) binding in CBS patients who present clinically with most affected right (R) side (*n* = 3) and patients who present clinically with most affected left (L) side (*n* = 8) compared to healthy controls. (B) Bar graph showing increases in [^18^F]AV1451 SUVR in the most, least affected side of patients with CBS and healthy controls. Whiskers indicate variability outside the upper and lower quartiles, the median is marked by a horizontal line inside the box. *P < 0.05; **P < 0.01. All *P* values are Bonferroni corrected for multiple comparisons. MA = most affected; LA = least affected

**Fig. 2:**
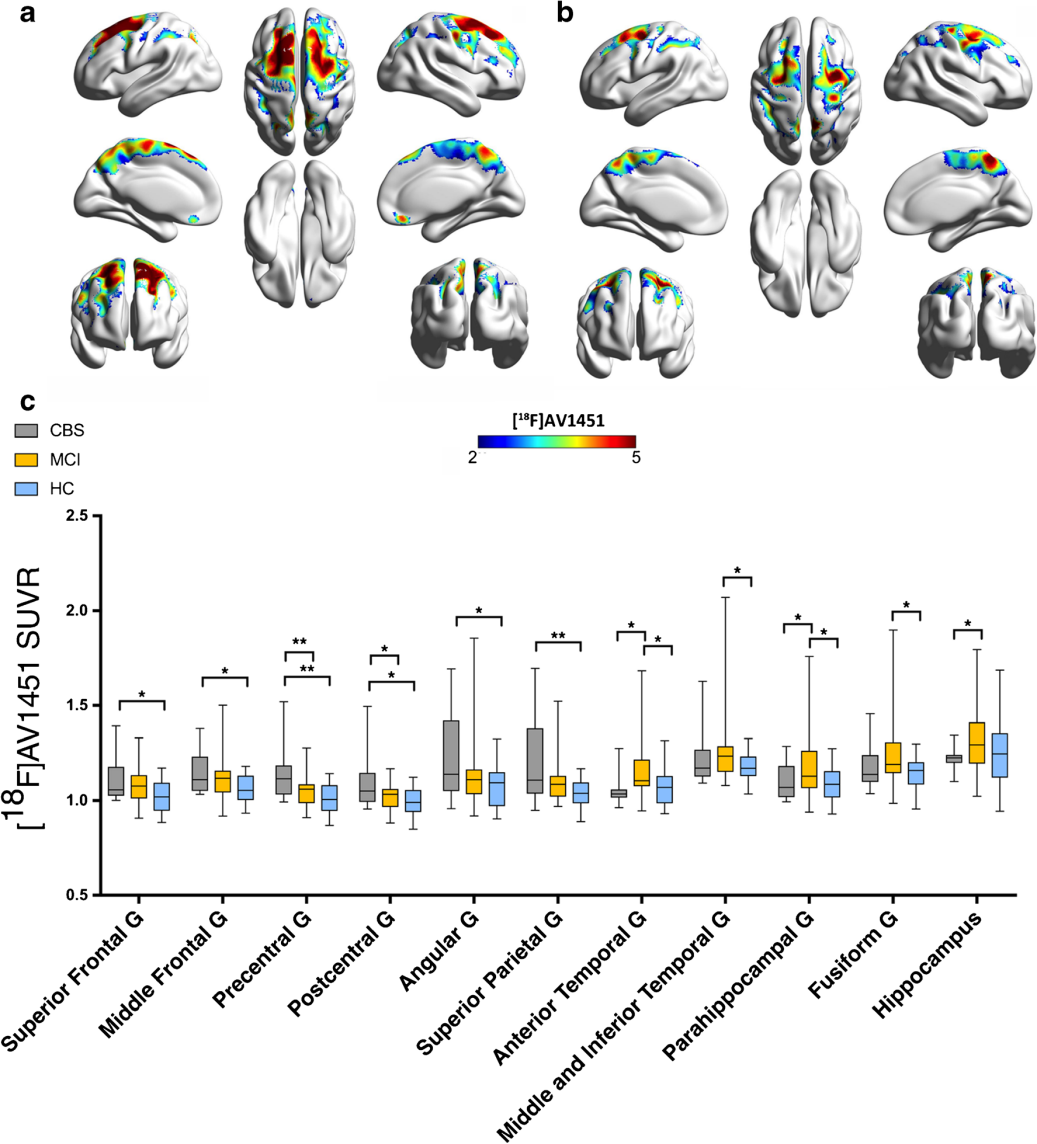
Increased tau deposition in anatomically defined brain regions of corticobasal syndrome patients compared to healthy controls and mild cognitive impairment patients. (A) Z-score maps showing increased [^18^F]AV1451 binding in CBS patients compared to healthy controls. (B) Z-score maps showing increased [^18^F]AV1451 SUVR in CBS patients compared to MCI patients. (C) Bar graph showing increases in [^18^F]AV1451 SUVR in patients with CBS most affected hemisphere, MCI and healthy controls. Whiskers indicate variability outside the upper and lower quartiles, the median is marked by a horizontal line inside the box. *P < 0.05. All *P* values are Bonferroni corrected for multiple comparisons

We found increases in cortical and subcortical [^18^F]AV1451 SUVRs in patients with CBS compared to the group of healthy controls (*P* < 0.05; Fig. 1, 2A, S2 and S3). Since asymmetric brain changes and clinical symptoms are features of CBS, we assessed tau deposition contralateral to the clinically most affected body side, compared to healthy controls and patients with MCI due to AD. We found differences in mean [^18^F]AV1451 SUVRs between the most and least affected hemispheres in the precentral gyrus (*P* = 0.047), postcentral gyrus (*P* = 0.044) and angular gyrus (*P* = 0.044) in our group of patients with CBS (Table 2; Fig. 1).

**Table 2:** [^18^F]AV1451 SUVR in anatomical brain regions in patients with corticobasal syndrome patients, mild cognitive impairment and healthy controls

Regions of Interest	HC (*n* = 20) (mean ± SD)	CBS MA (*n* = 11) (mean ± SD)	CBS LA (n = 11) (mean ± SD)	MCI (*n* = 33) (mean ± SD)	CBS vs. HC* *P* value	CBS vs. MCI** *P* value
Hippocampus	1.25 (±0.17)	1.22 (±0.06)	1.19 (±0.09)	1.30 (±0.16)	>0.10	0.016
Anterior Temporal gyrus	1.07 (±0.10)	1.06 (±0.13)	1.06 (±0.06)	1.16 (±0.22)	>0.10	0.007
Parahippocampal gyrus	1.09 (±0.09)	1.09 (±0.09)	1.07 (±0.13)	1.18 (±0.17)	>0.10	0.048
Superior Frontal gyrus	1.02 (±0.08)	1.12 (±0.15)	1.09 (±0.09)	1.08 (±0.10)	0.041	>0.10
Middle Frontal gyrus	1.06 (±0.07)	1.16 (±0.16)	1.11 (±0.07)	1.12 (±0.11)	0.031	>0.10
Precentral gyrus	1.01 (±0.07)	1.16 (±0.19)	1.10 (±0.11)	1.05 (±0.08)	0.007	0.008
Postcentral gyrus	0.99 (±0.07)	1.10 (±0.19)	1.01 (±0.14)	1.00 (±0.07)	0.033	0.034
Angular gyrus	1.07 (±0.11)	1.24 (±0.32)	1.18 (±0.20)	1.14 (±0.18)	0.037	>0.10
Superior Parietal gyrus	1.04 (±0.07)	1.20 (±0.25)	1.19 (±0.20)	1.10 (±0.12)	0.014	>0.10
Lateral Occipital Lobe	1.08 (±0.09)	1.22 (±0.29)	1.17 (±0.18)	1.16 (±0.14)	0.078	>0.10
Posterior Cingulate	1.08 (±0.09)	1.19 (±0.21)	1.17 (±0.15)	1.16 (±0.19)	0.057	>0.10
Posterior Temporal Lobe	1.12 (±0.06)	1.21 (±0.21)	1.18 (±0.14)	1.18 (±0.13)	0.091	>0.10
Superior Temporal gyrus	1.07 (±0.08)	1.13 (±0.20)	1.08 (±0.10)	1.11 (±0.08)	>0.10	>0.10
Middle and Inferior Temporal gyrus	1.18 (±0.08)	1.26 (±0.22)	1.21 (±0.13)	1.27 (±0.19)	>0.10	>0.10
Fusiform gyrus	1.15 (±0.09)	1.18 (±0.15)	1.16 (±0.10)	1.25 (±0.18)	>0.10	>0.10
Caudate	1.01 (±0.12)	0.99 (±0.10)	1.02 (±0.11)	1.05 (±0.09)	>0.10	>0.10
Putamen	1.39 (±0.11)	1.50 (±0.18)	1.48 (±0.17)	1.43 (±0.15)	0.037	>0.10
Globus Pallidus	1.55 (±0.14)	1.67 (±0.26)	1.66 (±0.28)	1.69 (±0.20)	>0.10	>0.10
Substantia Nigra	1.39 (±0.14)	1.32 (±0.18)	1.30 (±0.20)	1.38 (±0.16)	>0.10	>0.10

All *P* values are Bonferroni corrected for multiple comparisons. *P values for the most affected hemisphere of CBS patients vs healthy controls; *P values for the most affected hemisphere of CBS patients vs. MCI patients. CBS=corticobasal syndrome; HC = healthy controls; LA = least affected side; MA = most affected side; MCI = mild cognitive impairment; n = number of subjects

CBS patients had higher mean [^18^F]AV1451 SUVRs in the superior frontal gyrus (*P* = 0.041), middle frontal gyrus (*P* = 0.031), precentral gyrus (*P* = 0.007), superior parietal gyrus (*P* = 0.014), postcentral gyrus (*P* = 0.033), angular gyrus (*P* = 0.039) and putamen (*P* = 0.037) in the hemisphere contralateral to the clinically most affected side compared to the group of healthy controls (Table 2; Fig. 1). No differences were observed in mean [^18^F]AV1451 SUVRs in the globus pallidus, substantia nigra, temporal and occipital cortices of the most affected hemisphere compared to the healthy controls (all *P* > 0.05; Table 2).

MCI patients showed increases in [^18^F]AV1451 SUVRs in the anterior (*P* = 0.022), middle and inferior (*P* = 0.019) temporal lobe, parahippocampal gyrus (*P* = 0.019) and fusiform gyrus (*P* = 0.010) compared to the group of healthy controls (Fig. 2C). When comparing MCI and CBS patients, we found that CBS patients had increased [^18^F]AV1451 SUVRs in the precentral gyrus (*P* = 0.008) and postcentral gyrus (*P* = 0.034) in the hemisphere contralateral to the clinically most affected body side compared to the group of MCI patients (Table 2; Fig. 2B and C). Patients with MCI had increased [^18^F]AV1451 SUVRs in the hippocampus (*P* = 0.016), parahippocampal gyrus (*P* = 0.048) and anterior temporal gyrus (*P* = 0.007) compared with CBS patients (Table 2; Fig. 2B and C).

Whole brain voxel-wise analysis of [^18^F]AV1451 SUVRs between the group of CBS patients and healthy controls confirmed results from region of interest-based analysis. Whole brain analysis revealed clusters of significant increases in CBS patients in the middle and superior frontal cortex, dorsolateral frontal cortex, posterior medial frontal cortex, precentral gyrus, and postcentral gyrus (all *P* < 0.05; Table S5; Fig. S4A). Likewise, voxel-wise analysis showed clusters of significant increases in [^18^F]AV1451 SUVRs in the dorsolateral frontal cortex, parietal lobe and supramarginal gyrus of CBS patients compared to MCI patients (all *P* < 0.05; Table S5; Fig. S4B). Patients with MCI had clusters of significant increases in [^18^F]AV1451 SUVR in the superior, middle and inferior temporal gyrus and fusiform gyrus when compared to CBS patients (all *P* < 0.05; Table S5; Fig. S4C).

### [^18^F]AV45 PET findings

We found no differences in cortical and subcortical [^18^F]AV45 SUVRs between patients with CBS and the group of healthy controls (all *P* > 0.05; Fig. S1). Patients with MCI showed increased [^18^F]AV45 SUVRs in the hippocampus (*P* = 0.015), amygdala (*P* = 0.004), parahippocampal gyrus (*P* = 0.008), superior frontal gyrus (*P* = 0.014), middle frontal gyrus (*P* < 0.001), precentral gyrus (*P* < 0.001), postcentral gyrus (*P* < 0.001), angular gyrus (*P* = 0.01) and superior parietal gyrus (*P* < 0.001) compared to CBS patients (Table S6).

### Neuropathological results

**Fig. 3:**
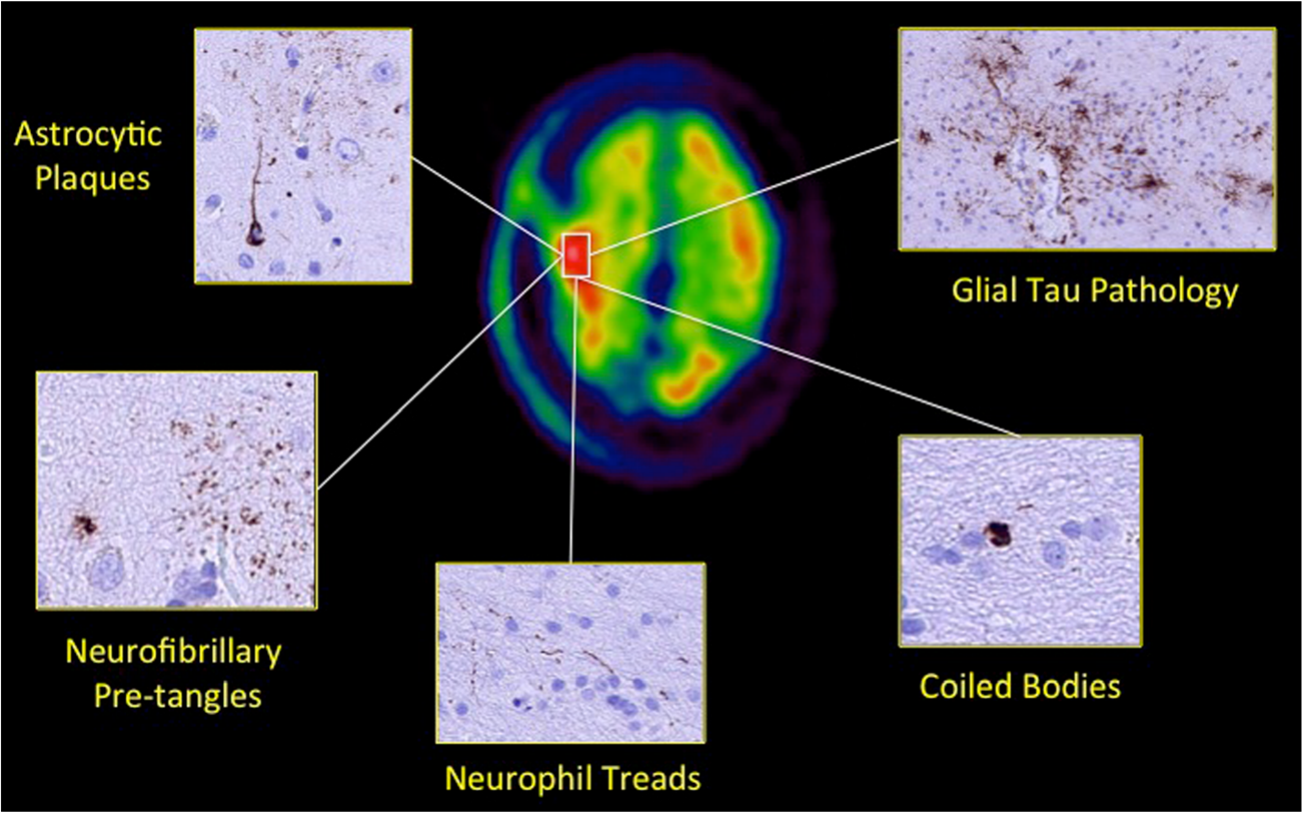
Histopathology evidence of increased tau deposition in a corticobasal syndrome patient. Axial summed [^18^F]AV1451 PET images fused co-registered and fused with 3 T MRI images for the cortex of a 75-year-old male CBS patient (CBS3; disease duration = 10 years; clinically most affected side = left; MMSE = 17; MoCA = 15; UPDRS-III = 63) who underwent brain biopsy showing increased right fronto-parietal [^18^F]AV1451 SUVR corresponding to the histopathological findings of subpial and perivascular glial tau pathology neuropil threads, rare coiled bodies, astrocytic plaques and neurofibrillary tangles and pre-tangles in neurones

Histopathology results from one CBS patient who underwent right frontal lobe biopsy for central nervous system lymphoma confirmed cortical tau deposition without amyloid-β parenchymal deposition. The tau pathology comprised sparse cortical pre-tangles and neurofibrillary tangles together with small numbers of neuropil threads. In addition, fine tau-positive processes with a plaque-like arrangement suggestive of astrocytic plaques were observed in the cortex in addition to sparse white matter threads and coiled bodies. Ubiquitin and p62 staining revealed neurofibrillary tangles and neuropil threads in the cortex. There was no alpha-synuclein pathology (Fig. 3).

### MRI findings

#### Volumetric findings

**Fig. 4:**
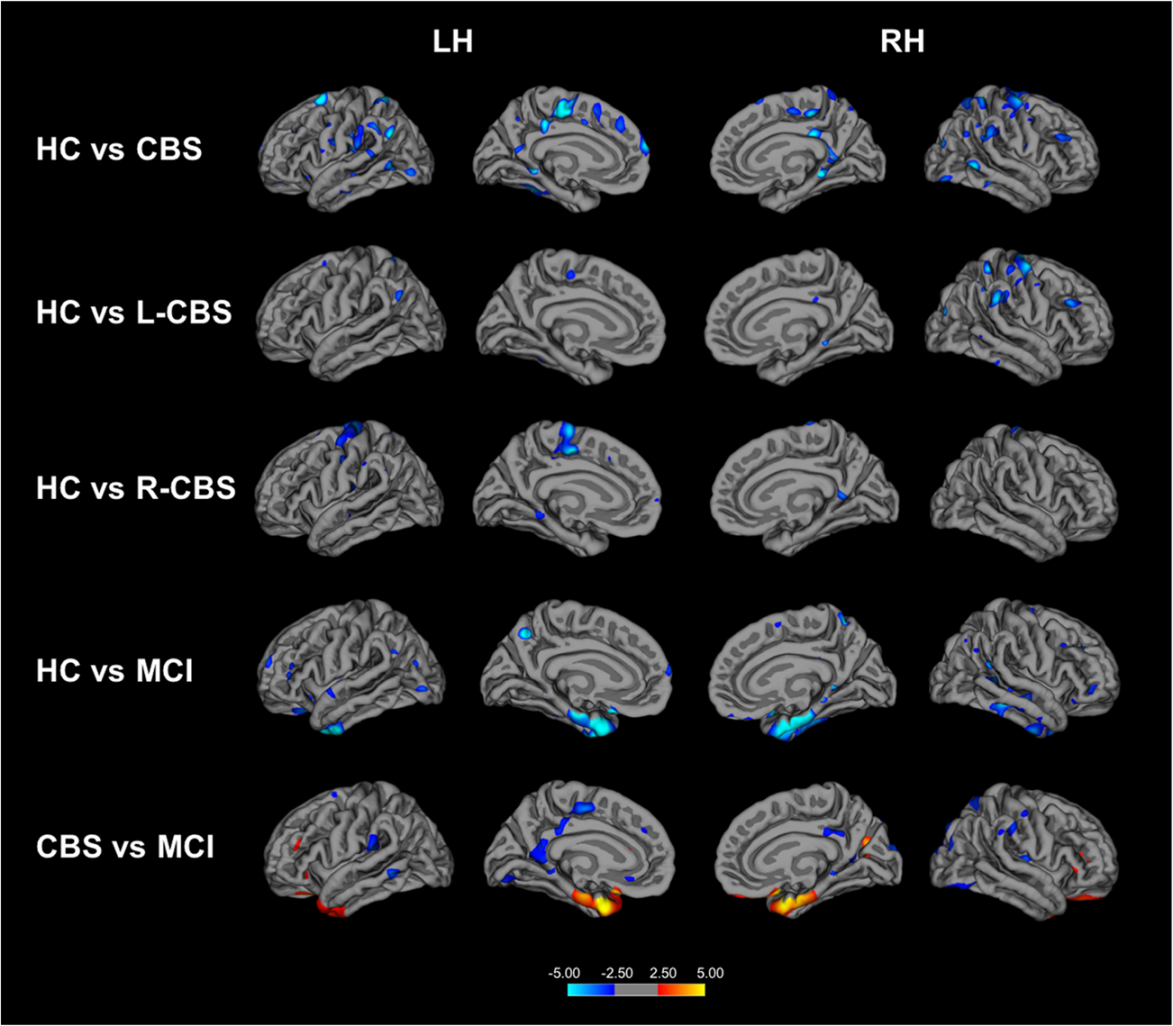
Volumetric changes in patients with corticobasal syndrome and mild cognitive impairment. Cortical areas showing decreased thickness in patients with CBS compared to healthy controls (top row); cortical thinning in CBS patients who present clinically with most affected left side (L-CBS; n = 8) (second row); and patients who present clinically with most affected right side (R-CBS; n = 3) (middle row). Cortical thinning in patients with MCI compared to healthy controls (fourth row). Cortical thickness in patients with MCI compared to CBS patients. Cortical thickness maps are displayed on average surface of FreeSurfer’s Qdec (Query, Design, Estimate and Contrast) interface. Colour bar indicated the Z scores. Results were obtained at *P* < 0.05 after multiple comparisons correction using Monte Carlo simulation. LH = Left Hemisphere; RH = Right Hemisphere; HC=Healthy Controls; CBS=Cortiobasal Syndrome; MCI = Mild Cognitive Impairment

FreeSurfer volumetric analysis showed decreased cortical thickness in the precentral gyrus (*P* = 0.019), supramarginal gyrus (*P* = 0.008) and middle frontal gyrus (*P* = 0.007) in the hemisphere contralateral to the clinically most affected body side of CBS patients compared to the group of healthy controls (Table S7; Fig. 4). When compared to MCI patients, CBS patients displayed decreases in cortical thickness in the middle frontal gyrus (*P* = 0.006), precentral gyrus (*P* = 0.009) and supramarginal gyrus (*P* = 0.006; Table S7; Fig. 4) in the hemisphere contralateral to the clinically most affected body side, whereas MCI patients showed cortical atrophy in temporal areas such as enthorinal cortex (*P* = 0.016) and temporal pole (*P* = 0.007) compared to CBS patients (Table S7, Fig. 4).

#### Microstructural white matter findings

**Fig. 5:**
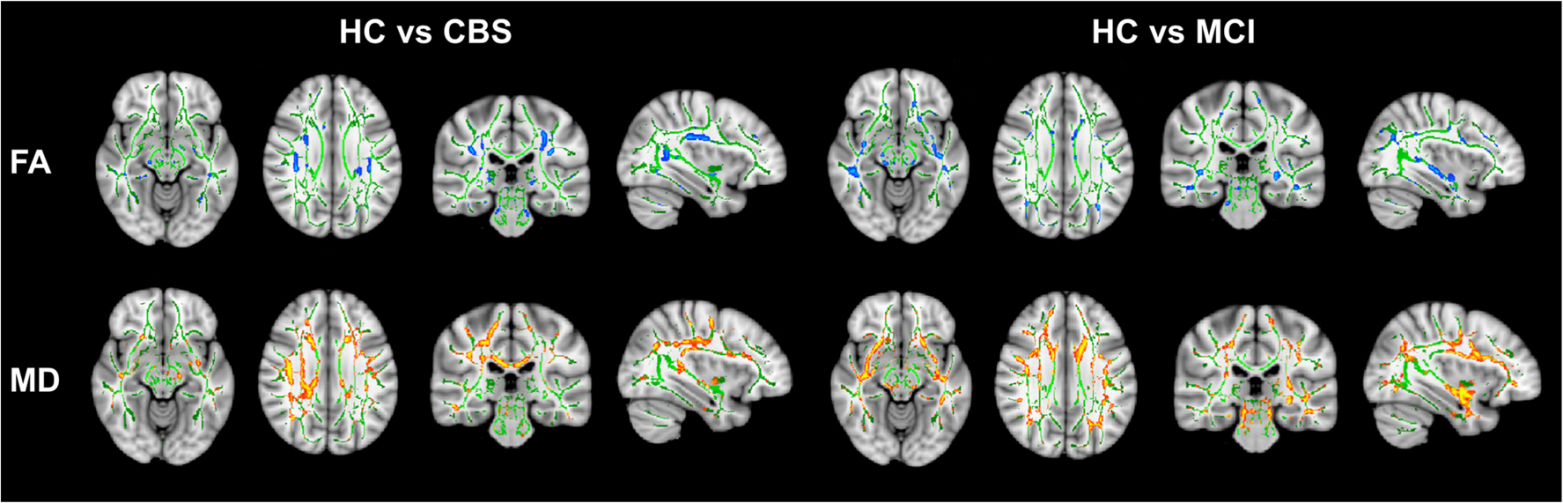
Microstructural white matter changes in patients with corticobasal syndrome and mild cognitive impairment compared to healthy controls. Tract-based spatial statistical maps of decreases in fractional anisotropy (FA) represented by blue voxels and increases mean diffusivity (MD) represented by red voxels. FA white matter skeleton is represented by green voxels. Results are reported after multiple comparison corrected at *P* < 0.05. MD = Mean Diffusivity; FA = Fractional Anisotropy; CBS=Corticobasal Syndrome; MIC = Mild Cognitive Impairment

Diffusion tensor imaging showed decreased FA in the angular gyrus (*P* = 0.008), precentral gyrus (*P* = 0.037), superior frontal gyrus (*P* = 0.039) and superior parietal gyrus (*P* = 0.035) and increased MD in the angular gyrus (*P* = 0.007), precentral gyrus (*P* = 0.018), middle frontal gyrus (*P* = 0.013), postcentral gyrus (*P* = 0.001) and superior parietal gyrus (*P* = 0.001) in the hemisphere contralateral to the clinically most affected body side of CBS patients compared to the group of healthy controls (Table S8; Fig. 5). When compared to MCI patients, CBS patients showed increases in MD in the precentral gyrus (*P* = 0.042), postcentral gyrus (*P* = 0.020), superior parietal gyrus (*P* = 0.034) and supramarginal gyrus (*P* = 0.002; Table S8; Fig. 5) in the hemisphere contralateral to the clinically most affected body side. No differences were observed in FA values between CBS and MCI patients (all *P* > 0.05; Table S8; Fig. 5).

We repeated the PET and MRI analysis by co-varying between data acquired at our centre and the ADNI dataset and we found no differences in our results.

### Correlations

We found a significant negative correlations between decreased cortical thickness in the precentral gyrus in the hemisphere contralateral to the clinically most affected body side and motor performance scores on the finger tapping (UPDRS-III Item 3.4; *r*_*s*_ = −0.86; *P* = 0.001), hand movements (UPDRS-III Item 3.5; *r*_*s*_ = −0.78; *P* = 0.008), pronation/supination movements of the hand (UPDRS-III Item 3.6; *r*_*s*_ = −0.71; *P* = 0.022) and apraxia of hand movement (PSPRS Item 22; *r*_*s*_ = −0.68; *P* = 0.031) of the clinically most affected side in our group of CBS patients (Fig. S5A).

MD values in the precentral gyrus in the hemisphere contralateral to the clinically most affected body side correlated positively with motor scores for finger tapping movements (UPDRS-III Item 3.4; *r*_*s*_ = 0.81; *P* = 0.027), hand movements (UPDRS-III Item 3.5; *r*_*s*_ = 0.81; *P* = 0.027), pronation/supination movements of the hand (UPDRS-III Item 3.6; *r*_*s*_ = 0.82; *P* = 0.024) and apraxia of hand movement (PSPRS Item 22; *r*_*s*_ = 0.87; *P* = 0.010) of the clinically most affected body side in our group of CBS patients (Fig. S5B). We also detected a negative correlation between FA values in the precentral gyrus in the hemisphere contralateral to the clinically most affected body side and upper limb rigidity movements (UPDRS-III Item 3.3; *r*_*s*_ = −0.80*; P* = 0.031) of the clinically most affected body side (Fig. S6).

Finally, performance on the Rapid Visual Information Processing (RVP) test correlated negatively with [^18^F]AV1451 SUVR in middle frontal gyrus (*r*_*s*_ = −0.79; *P* = 0.036) and postcentral gyrus (*r*_*s*_ = −0.79; *P* = 0.036) in the hemisphere contralateral to the clinically most affected body side in our group of CBS patients (Fig. S7).

We did not find any significant correlations between cortical [^18^F]AV1451 SUVRs and clinical symptoms.

## Discussion

Our findings demonstrate the presence of frontal and parietal tau and microstructural pathology, in the absence of amyloid-β pathology, in the affected hemisphere contralateral to the clinically most affected side of patients with CBS. Our findings derive from in vivo assessments of molecular and structural pathology following PET and MRI, which are consistent with observations from histopathological studies [2]. We also present one case, who underwent both the in vivo imaging study and histopathological examination of brain biopsy, and confirmed co-localisation of increased PET tau signal and tau pathology in the parietal cortex of the affected hemisphere contralateral to the clinically most affected side providing with additional validation of our findings.

Our study follows three recent pilot studies which assessed tau pathology with either the same [^18^F]AV1451 PET radioligand [8, 9] we used, or with the [^18^F]THK5351 PET radioligand [26]. Our findings are in line and extend the preliminary observations from these studies that showed frontal and parietal tau pathology in brain areas including the precentral, postcentral and superior frontal and superior parietal gyri in patients with CBS. These previous studies, however, have been limited in scope due to limited sample size and not assessing some other important elements of pathology such as grey and white matter microstructural changes. Our study comes with the significant advantages that our group of patients with CBS was double the size of that used in previous pilot studies; the depth of assessments including thorough clinical and neurophysiological evaluation, and multimodal tau and amyloid-β molecular and volumetric and microstructural assessment of molecular and structural pathology in vivo; the comparisons with large sized cohorts of healthy individuals, but also patients with MCI due to AD, and in one case the concurrent tau and amyloid-β PET imaging and histopathological examination of brain biopsy.

Another advantage of our study was to validate SUVRs against arterial input function method for quantification of [^18^F]AV1451 in vivo. To validate a suitable reference region for use in simplified models, full arterial quantification of [^18^F]AV1451 was carried out using the 2-TC model for estimation of V_T_; no difference was found in the reference region V_T_ between groups. Therefore, reference region was used to quantified [^18^F]AV1451 using the indirect Logan DVR and SUVR [14, 27]. Indirect Logan DVR measures were derived from compartmental modelling with arterial inputs, namely V_T_^tissue/^V_T_^ref^. [^18^F]AV1451 uptake is most commonly measured using semi-quantitative SUVRs [28–30] with the cerebellum as the reference region for no tau-specific [^18^F]AV1451 uptake [19]. SUVRs have several advantages over computational analysis with plasma input functions, including shorter scan duration, with static scans targeting a specific time window, reduced likelihood of head movement and simplified and quick analysis method. Furthermore, quantitative of static imaging with SUVRs static imaging has greater potential for clinical applications. Here, we show no differences in results at a group level when using SUVR or Logan DVR values. Therefore, supporting previous work [14, 27], [^18^F]AV1451 can be analysed without the need for arterial sampling and compartmental modelling. Static imaging with SUVRs provides a reliable method for the regional quantification of tau burden in patients with CBS.

The region-of-interest analysis we performed showed increases in tau deposition in the superior frontal gyrus, middle frontal gyrus, precentral gyrus, superior parietal gyrus, postcentral gyrus, angular gyrus and putamen in the hemisphere contralateral to the clinically most affected side. These findings were also confirmed at voxel level. Moreover, we found that increases in cortical tau pathology co-localised with cortical grey matter loss and white matter microstructural changes. It is likely that abnormal accumulation of hyperphosphorylated 4R tau may cause neuronal loss and white matter axonal loss. Tau pathology is also found in white matter as neuropil threads and oligodendroglial coiled bodies in CBS postmortem tissue [2]. Smith et al. suggested that cortical atrophy is more pronounced and widespread compared to cortical [^18^F]AV1451 deposition in CBS patients [8]. However, this observation was not confirmed in our larger group of CBS patients. Moreover, it may be possible that the amount of tau pathology visualised with [^18^F]AV1451 is lower than expected because of the low affinity of this radioligand for 4R tau protein.

It has been suggested that [^18^F]AV1451 selectively binds to paired helical filaments 3R characteristic of AD and less avidly to the straight tau filaments 4R typical of non-AD tauopathies such as CBS and PSP [5, 6]. Our histopathological data, however, support that the cortical increases observed in [^18^F]AV1451 uptake corresponded to abnormal accumulation of hyperphosphorylated 4R tau in neurons and in glial cells. In support of our findings, previous neuropathological studies have shown that [^18^F]AV1451 uptake correlates with 4R-tau burden in autopsy-confirmed CBS post-mortem tissue [31, 32]. Increases in midbrain and basal ganglia [^18^F]AV1451 uptake were also shown found in other 4R tauopathies such as PSP [33–35] and in MAPT p.R406W mutation carriers [36].

CBS pathology affects also subcortical nuclei such as striatum, globus pallidus and substantia nigra [2]. We found significant increases in tau deposition in the putamen in the hemisphere contralateral to the most affected side in CBS patients. Neuropathological and autoradiographic data have suggested that [^18^F]AV1451 exhibits off-target binding to neuromelanin- and melanin-containing neurons in subcortical nuclei [5]. However, a recent [^18^F]AV1451 PET study showed increased uptake in the basal ganglia and midbrain of PSP patients in absence of post-mortem neuromelanin-containing cells [34]. Given that this is still a subject of debate we will not provide interpretation and mechanistic speculation about our findings in putamen.

In our study, we compared imaging data from the group of patients with CBS to a group of patients with MCI due to AD, in addition to the group of healthy controls. The patients with MCI showed significant tau retention in the anterior, middle, inferior temporal lobe, parahippocampal gyrus and fusiform gyrus compared to the group of healthy controls. These findings reflect the distribution of tau pathology consistent with Braak stage III-IV, which involves hippocampus and the anterior part of the temporal lobe [37]. Compared to patients with CBS, patients with MCI displayed significant increases in tau deposition in the hippocampus, parahippocampal gyrus and anterior temporal gyrus; whereas patients with CBS showed increases in tau deposition in precentral and postcentral gyri in the affected hemisphere. This suggests different disease-specific patterns of tau pathology in CBS patients and MCI patients, with the former involving the primary motor and primary somatosensory cortices of the hemisphere contralateral to the clinically affected side of the body.

All our CBS patients had normal cortical and subcortical amyloid-β retention indicating the absence of typical AD pathology. This was also confirmed in the case of the patient with CBS who underwent histopathological examination of brain biopsy. As expected, MCI patients showed increased amyloid-β deposition across several temporal and parietal areas consistent with previous studies [38].

We found that increased tau deposition in the medial frontal and postcentral gyri contralateral to the clinically most affected side was associated with worse performance at the Rapid Visual Information Processing test, which measures attention. The medial frontal cortex plays a key role in performance monitoring on subsequent trials and in the implementation of associated adjustments in cognitive control [39], whereas the somatosensory area has been commonly involved in the execution of visual motor task, which require sustained attention [40]. A recent in vivo [^18^F]AV1451 PET study showed that increased tau uptake in the precentral grey and white matter was associated with worse motor functions as measured by the UPDRS-III and this correlation was drive by bradykinesia and axial motor subscores [9]. We did not find associations between motor symptoms severity and increased tau deposition. This discrepancy may be due to the small sample size investigated by Cho et al., [9] who interrogated correlations between tau and clinical symptoms only in six CBS patients. Moreover, the lack of a validated scale to assess motor symptoms in CBS may have also contributed to this difference.

MRI analysis showed disease-related patterns of grey and white matter changes in CBS and MCI patients. We found significant grey matter loss in the precentral, supramarginal and middle frontal gyri in the hemisphere contralateral to the clinically most affected body side of the patients with CBS compared to healthy controls and patients with MCI. Microstructural white matter changes were also observed in frontal and parietal cortices in the hemisphere contralateral to the clinically most affected body side of patients with CBS compared to healthy controls and patients with MCI. This is in line with previous studies showing significant asymmetric regional grey matter loss and white matter changes in motor cortex areas [10, 11].

We found significant associations between grey matter loss and white matter changes in the precentral gyrus in the hemisphere contralateral to the clinically most affected side and hand rigidity, bradykinesia and apraxia of the affected clinical body side. The clinical core features of CBS include asymmetric rigidity, bradykinesia and apraxia characteristically affecting the upper limbs [41]. This suggests that grey and white matter structural changes in the primary motor cortex are associated with worse clinical symptoms in CBS. We measured motor symptoms severity using both the UPDRS-III and PSPRS since to date there is not a validate clinical rating scale for CBS.

In conclusion, our findings demonstrate the identification of an in vivo disease-related pattern of asymmetric frontal and parietal tau and microstructural pathology in the absence of amyloid-β, which distinguishes CBS from non-affected individuals and patients with MCI due to AD. Our results are confirmed at a histopathological level and support the use of [^18^F]AV1451 PET as a marker of tau pathology in CBS patients. Clinical diagnosis of CBS could be difficult due to the overlapping features with other neurodegenerative disorders, in vivo imaging of tau aggregates with PET has the potential to aid in the differential diagnosis of CBS. Since also prevention of tau aggregation and propagation is the focus of attempts to develop mechanism-based treatments for tauopathies our multimodal image approach could also serve as an indicator of treatment efficacy for interventions aimed at preventing tau aggregate formation. Further studies are needed to demonstrate changes in [^18^F]AV1451 PET and microstructure over time and to establish their full potential as biomarkers to stratify and monitor the effect of disease-modifying drugs in future clinical trials.

## Electronic supplementary material

ESM 1 (DOCX 5528 kb)

## Abbreviations

DVR: Distribution Volume Ratio
MRI: Magnetic Resonance Imaging
PET: Positron Emission Tomography
PSPRS: Progressive Supranuclear Palsy Rating Scale
SUVR: Standardised Uptake Volume Ratio
UPDRS: Unified Parkinson’s Disease Rating Scale
