# Supplementary material for: Disease-related patterns of in vivo pathology in Corticobasal syndrome
Source: Eur J Nucl Med Mol Imaging. 2018 Aug 8;45(13):2413–25. doi: 10.1007/s00259-018-4104-2 (PMC6208819; doi:10.1007/s00259-018-4104-2)
Supplement: Supplementary file 1 — (DOCX 5528 kb) [file 259_2018_4104_MOESM1_ESM.docx]

**SUPPLEMENTAL MATERIAL**

**Disease-related patterns of *in vivo* pathology in Corticobasal Syndrome**

Flavia Niccolini,^1*^ Heather Wilson,^1*^ Stephanie Hirschbichler,^2^ Tayyabah Yousaf_,_^1^ Gennaro Pagano,^1^ Alexander Whittington,^3^ Silvia Paola Caminiti,^1^ Roberto Erro,^4^ Janice L Holton,^5^ Zane Jaunmuktane,^5^ Marcello Esposito,^6^ Davide Martino,^7^ Ali Abdul,^8^ Jan Passchier,^8^ Eugenii A. Rabiner,^8,9^ Roger N, Gunn,^3,8^ Kailash P. Bhatia,^2^ and Marios Politis^1^ for the Alzheimer’s Disease Neuroimaging Initiative^**^

^1^Neurodegeneration Imaging Group, Institute of Psychiatry, Psychology and Neuroscience, King’s College London, London, UK

^2^Sobell Department of Motor Neuroscience, UCL Institute of Neurology, London, UK

^3^Division of Brain Sciences, Department of Medicine, Imperial College London, London, UK

^4^Center for Neurodegenerative Diseases (CEMAND) Department of Medicine, Surgery and Dentistry, University of Salerno, Italy

^5^Division of Neuropathology, UCL Institute of Neurology, London, UK

^6^Department of Neurosciences, Reproductive Sciences and Odontostomatology, Federico II University of Naples, Italy

^7^Department of Clinical Neurosciences, Cumming School of Medicine, University of Calgary, Calgary, Canada

^8^Imanova Ltd, Centre for Imaging Sciences, Hammersmith Hospital, London, UK

^9^Centre for Neuroimaging Sciences, Institute of Psychiatry, Psychology and Neuroscience, King s College London, London, UK

*These authors contributed equally

*Correspondence & reprint* requests to Professor Marios Politis, Neurodegeneration Imaging Group, Maurice Wohl Clinical Neuroscience Institute, Institute of Psychiatry, Psychology and Neuroscience (IoPPN), King’s College London, 125 Coldharbour Lane, Camberwell, London SE5 9NU, UK. E-mail: marios.politis@kcl.ac.uk.

**Some of the data used in preparation of this article were obtained from the Alzheimer’s Disease Neuroimaging Initiative (ADNI) database (adni.loni.usc.edu). As such, the investigators within the ADNI contributed to the design and implementation of ADNI and/or provided data but did not participate in analysis or writing of this report. A complete listing of ADNI investigators can be found at: http://adni.loni.usc.edu/wpcontent/uploads/how_to_apply/ADNI_Acknowledgement_List.pdf.

**TABLE OF CONTENT**

SUPPLEMENTAL METHODS 3

Clinical assessments 3

Imaging assessments 3

Region of interest-based analysis 4

Voxel-wise analysis 5

Neuropathological analysis 5

SUPPLEMENTAL RESULTS 5

Table S1 5

Table S2 6

Table S3 7

Table S4 8

Table S5 10

Table S6 11

Table S7 11

Table S8 12

Figure S1 14

Figure S2 15

Figure S3 16

Figure S4 17

Figure S5 18

Figure S6 19

Figure S7 19

REFERENCES 20

# SUPPLEMENTAL METHODS

## Clinical assessments

Motor symptom severity was assessed with the Unified Parkinson’s Disease Rating Scale part-III (UPDRS-III) and PSP Rating Scale (PSPRS) part-III, IV, V and VI. Neuropsychiatric symptoms were assessed with the Neuropsychiatric Inventory (NPI), Geriatric Depression Scale (GDS), and the Hamilton Depression Rating Scale (HDRS). Frontal symptoms were assessed with the Frontal Assessment Battery (FAB). The Mini Mental Status Examination (MMSE), PSPRS part-II and Montreal Cognitive Assessment (MoCA) were used to assess general cognitive status. Further cognitive assessments were carried out using the Cambridge Neuropsychological Test Automated Battery (CANTAB^®^) and included assessments related to psychomotor speed (Reaction Time), attention (Rapid Visual Information Processing), episodic memory (Paired Associate Learning and Delayed Match to Sample), working memory and executive function (Spatial Working Memory). Global non-motor symptoms burden was assessed with the UPDRS part I and the Scales for Outcomes in Parkinson's disease–Autonomic (SCOPA-AUT) and excessive daytime sleepiness with the Epworth Sleepiness Scale (ESS). Functional capacity was assessed with the Activities of Daily Living (ADL), Instrumental Activities of Daily Living (IADL) and UPDRS part II.

## Imaging assessments

CBS patients and five healthy controls underwent positron emission tomography (PET) and magnetic resonance imaging (MRI), which was performed at Imanova Ltd, London, UK. Participants were scanned on a Siemens Biograph Hi-Rez 6 PET-CT scanner (Erlangen, Germany). A mean dose of 164.82 MBq (SD: ±5.4) [^18^F]AV1451 [mean mass injected: 0.14 ug (SD: ±0.1)] was administered intravenously as a slow bolus injection over 20s. A mean dose of 155.83 MBq (SD: ±11.5) [^18^F]AV45 [mean mass injected: 0.20 ug (SD: ±0.2)] was administered intravenously as a slow bolus injection over 10s and followed by a flush of approximately 10 mL of sodium chloride 9 mg/mL (0.9%) solution for injection to ensure full delivery of the dose. Dynamic emission data were acquired continuously for 120 minutes following the injection of [^18^F]AV1451 and 60 minutes following the injection of [^18^F]AV45. The dynamic images were reconstructed into 26 frames (8 x 15 s, 3 x 60 s, 5 x 120 s, 5 x 300 s, and 5 x 600 s), using a filtered back projection algorithm (direct inversion Fourier transform) with a 128 matrix, zoom of 2.6 producing images with isotropic voxel size of 2 x 2 x 2 mm^3^, and smoothed with a transaxial Gaussian filter of 5 mm. Blood sampling was performed for [^18^F]AV1451 through an arterial line inserted in the radial artery to generate arterial plasma input data. For the initial 15 minutes radioactivity levels in blood was continuously measured through an automatic blood sampling system at 5ml/min, followed by samples at 5, 10, 20, 40, 50, 60, 80 and 100 min during the scan.

Participants from the ADNI database, fifteen healthy controls and thirty-three MCI patients, underwent an [^18^F]AV1451 scan, [^18^F]AV45 scan, and structural T1-weighted MRI and DTI scan. PET imaging was performed at each ADNI site according to standardized acquisition protocols; PET scans underwent quality control protocol and processed to produce final images with standard orientation, voxel size and 8 mm^3^ resolution [1]. Participants underwent a static [^18^F]AV1451 scan 75-105 minutes after injection (6 x 5 minute frames) with a mean dose of 376 MBq (SD: ±31.9) [^18^F]AV1451, and a static [^18^F]AV45 scan 50-60 minutes after injection with a mean dose of 3701 MBq (SD: ±23.9) [^18^F]AV45. We tested compatibility of data from the two acquisition protocols and found little difference in [^18^F]AV1451 standardised uptake value ratio (SUVR) values from SUVR_75-105_ and SUVR_60-80_ between the healthy control cohorts.

MRI scans were acquired with a 32-channel head coil on a Siemens Magnetom MAGNETOM TrioTim syngo MR B17 (Erlangen, Germany), 3T MRI scanner. Structural MRI for participants from the ADNI database, were acquired at ADNI-2 sites equipped with 3T MRI scanners using a 3D MPRAGE T1-weighted sequences with sagittal slices and voxel size of 1.1 x 1.1 x 1 .2 mm^3^, as described online (http://adni.loni. usc.edu/methods/documents/mri-protocols).

### *Region of interest-based analysis*

The multi-atlas propagation with enhanced registration (MAPER) was used to define region of interests.^2^ MRI scans were automatically segmented using the MAPER approach into 95 anatomic regions. This robust technique improves the quality of multi-atlas based automatic whole-brain segmentations [2], allows the automatic segmentation of PET data in anatomic regional volumes, and is applicable even to subjects with significant cortical atrophy and ventriculomegaly. The output object maps were visually checked independently by two reviewers to ensure accurate segmentation based on each subjects’ structural MRI.

[^18^F]AV1451 and [^18^F]AV45 PET images of each subject were co-registered to their respective structural MRI using SPM12 (Wellcome Trust Centre for Neuroimaging) in MATLAB (r2015a; The MathWorks). The quality of co-registration was checked visually for each subject. The subjects’ individual 95 regions of interest (ROIs) were overlaid on subjects’ PET data which was sampled with the ANALYZE medical imaging software (version 12, Mayo Foundation AnalyzeDirect) to extract uptake values within regions-of-interest.

### *Voxel-wise analysis*

Spatial processing and statistical analysis were performed using SPM12 implemented in Matlab 2015a. T1 weighted MR images were spatially normalized to the T1 MNI template and the transformation parameters applied to [^18^F]AV1451 SUVR maps. [^18^F]AV1451 SUVR images were smoothed by application of 8mm full-width at half maximum Gaussian kernel. Voxel-wise statistics for between-group comparisons were computed using the two-sample t-test implemented in SPM12, with age as covariate, applying appropriately weighted contrasts to localise significant increases in mean voxel [^18^F]AV1451 SUVR. The threshold for statistical significance was set to *P*<0.05, at a cluster level, after family wise error (FWE) correction for multiple comparisons. Individual z-score maps were also derived for [^18^F]AV1451 SUVR uptake in each CBS patient compared to cohort of healthy controls; z-score maps were derived on a voxel basis using the general linear model [3].

### *Neuropathological analysis*

One patient underwent cerebral frontal cortex biopsy for central nervous system lymphoma. Histological sections were stained using routine histological (haematoxylin and eosin, H&E) technique. Immunohistochemistry using antibodies to the following proteins was performed using a standard avidin–biotin method: tau (AT8 clone; Thermo scientific MN1020; 1:600), amyloid-β (Aβ; Biosource international, Mouse Dako, clone 6F/3D; 1:100) and alpha-synuclein (Vector Laboratories; KM51; 1:50).

# SUPPLEMENTAL RESULTS

## Table S1 Clinical assessments of patients with Corticobasal Syndrome and Healthy Controls.

|  | Healthy Controls | CBS patients | *P* value* |
| --- | --- | --- | --- |
| Motor Assessments | | | |
| UPDRS-III (mean ±SD) | 0.00 (±0.0) | 50.91 (±27.7) | <0.001 |
| PSPRS-III (mean ±SD) | 0.00 (±0.0) | 2.36 (±2.8) | 0.06 |
| PSPRS-IV (mean ±SD) | 0.00 (±0.0) | 4.27 (±3.3) | 0.007 |
| PSPRS-V (mean ±SD) | 0.00 (±0.0) | 8.64 (±3.9) | <0.001 |
| PSPRS-VI (mean ±SD) | 0.00 (±0.0) | 10.64 (±7.7) | 0.005 |
| Cognitive Assessments | | | |
| MMSE (mean ±SD) | 29.67 (±0.8) | 23.64 (±5.4) | 0.017 |
| MOCA (mean ±SD) | 29.33 (±1.6) | 17.82 (±6.5) | <0.001 |
| FAB (mean ±SD) | 17.67 (±0.8) | 8.60 (±4.4) | <0.001 |
| PSPRS-II (mean ±SD) | 0.00 (±0.00) | 2.82 (±2.8) | 0.008 |
| Neuropsychiatric Assessments | | | |
| NPI (mean ±SD) | 0.00 (±0.00) | 4.82 (±4.1) | 0.013 |
| GDS (mean ±SD) | 1.50 (±1.9) | 11.78 (±9.6) | 0.024 |
| HDRS (mean ±SD) | 0.50 (±0.8) | 8.73 (±5.6) | 0.003 |
| Non-motor Symptom Assessments | | | |
| UPDRS-I (mean ±SD) | 1.17 (±1.6) | 13.00 (±8.4) | 0.004 |
| SCOPA-AUT (mean ±SD) | 4.67 (±3.8) | 14.64 (±7.4) | 0.008 |
| ESS (mean ±SD) | 2.50 (±2.3) | 6.82 (±4.4) | 0.040 |
| Functional Assessments | | | |
| ADL (mean ±SD) | 6.00 (±0.0) | 3.00 (±2.7) | 0.016 |
| IADL (mean ±SD) | 8.00 (±0.0) | 4.36 (±3.2) | 0.015 |
| UPDRS-II (mean ±SD) | 0.00 (±0.0) | 26.45 (±15.0) | <0.001 |

ADL=Activities of Daily Living; CDR= Clinical Dementia Rating scale; ESS=Epworth Sleepiness Scale; FAB=Frontal Assessment Battery; GDS=Geriatric Depression Scale; HDRS=Hamilton Depression Rating Scale; IADL=Instrumental Activities of Daily Living; MMSE=Mini Mental Status Examination; MoCA=Montreal Cognitive Assessment; NPI=Neuropsychiatric Inventory; PSPRS=Progressive Supranuclear Palsy Rating Scale; SCOPA-AUT=Scales for Outcomes in Parkinson's disease–Autonomic; UPDRS=Unified Parkinson’s Disease Rating Scale. *All *P* values are Bonferroni corrected for multiple comparisons.

## Table S2 Cognitive assessments in the groups of Corticobasal Syndrome patients and healthy controls.

| Cognitive Assessments | Healthy Controls | | Corticobasal Syndrome patients | |
| --- | --- | --- | --- | --- |
| *Psychomotor speed* | | | | |
| RTI median reaction time (simple) (±SD) [msec] | 382.58 (±30.1) |  | | 624.00 (±291.1) |
| RTI median movement time (simple) (±SD) [msec] | 205.08 (±89.0) |  | | 450.63 (±265.6) |
| RTI median reaction time (five-choice) (±SD) [msec] | 440.75 (±30.8) |  | | 641.88 (±160.0)* |
| RTI median movement time (five-choice) (±SD) [msec] | 239.17 (±65.4) |  | | 52.63 (±265.6)* |
| *Attention* | | | | |
| RVP A-time (±SD) | 0.86 (±0.0) |  | | 0.78 (±0.1)* |
| RVP median latency (±SD) [msec] | 494.50 (±64.0) |  | | 800.50 (±2.16.0)** |
| *Episodic memory* | | | | |
| PAL tot errors (±SD) | 22.17 (±15.9) |  | | 43.88 (±20.4) |
| DMS % correct (±SD) | 83.33 (±8.2) |  | | 60.63 (±23.5)* |
| DMS median correct latency (±SD) [msec] | 3719.25 (±1004.0) |  | | 4939.94 (±2525.6) |
| DMS probability of given error (±SD) | 0.09 (±0.1) |  | | 0.41 (±0.2)** |
| *Working memory and executive function* | | | | |
| SWM between errors (±SD) | 21.00 (±2.7) |  | | 21.13 (±8.7) |
| SWM strategy (±SD) | 9.83 (±1.5) |  | | 9.13 (±2.1) |

DMS: Delayed match to sample; PAL: Paired Associates Learning; RTI: Reaction Time Test; RVP: Rapid Visual Information Processing Test; SWM: Spatial Working Memory. **P* <0.05, ***P* <0.01. All *P* values are Bonferroni corrected for multiple comparisons.

## Table S3 [^18^F]AV1451 Logan DVR and SUVR values in anatomical brain regions in the group of Corticobasal Syndrome patients.

| Regions of Interest | Logan DVR (mean±SD) | SUVR (mean±SD) |
| --- | --- | --- |
| Frontal Lobe | | |
| Superior Frontal gyrus | 1.05 (±0.08) | 1.10 (±0.12) |
| Middle Frontal gyrus | 1.07 (±0.08) | 1.14 (±0.11) |
| Inferior Frontal gyrus | 1.07 (±0.07) | 1.13 (±0.10) |
| Precentral gyrus | 1.07 (±0.11) | 1.14 (±0.15) |
| Parietal Lobe | | |
| Angular gyrus | 1.15 (±0.20) | 1.21 (±0.25) |
| Superior Parietal gyrus | 1.14 (±0.18) | 1.19 (±0.23) |
| Postcentral gyrus | 1.03 (±0.14) | 1.08 (±0.16) |
| Supramarginal gyrus | 1.09 (±0.21) | 1.14 (±0.20) |
| Occipital Lobe | | |
| Lateral Occipital Lobe | 1.13 (±0.18) | 1.19 (±0.22) |
| Lingual gyrus | 1.07 (±0.09) | 1.10 (±0.12) |
| Cuneus | 1.07 (±0.09) | 1.10 (±0.14) |
| Temporal Lobe | | |
| Posterior Temporal Lobe | 1.13 (±0.14) | 1.19 (±0.17) |
| Superior Temporal gyrus | 1.06 (±0.10) | 1.11 (±0.15) |
| Middle and Inferior Temporal gyrus | 1.15 (±0.13) | 1.23 (±0.17) |
| Fusiform gyrus | 1.07 (±0.10) | 1.17 (±0.13) |
| Anterior Cingulate | 0.97 (±0.07) | 1.04 (±0.10) |
| Posterior Cingulate | 1.16 (±0.15) | 1.22 (±0.24) |
| Subcortical brain regions | | |
| Amygdala | 1.07 (±0.13) | 1.16 (±0.14) |
| Brainstem | 0.86 (±0.09) | 0.937 (±0.10) |
| Caudate | 0.94 (±0.11) | 1.00 (±0.08) |
| Putamen | 1.42 (±0.14) | 1.49 (±0.17) |
| Globus Pallidus | 1.55 (±0.25) | 1.66 (±0.27) |
| Thalamus | 1.18 (±0.14) | 1.23 (±0.14) |
| Nucleus Accumbens | 1.17 (±0.10) | 1.26 (±0.13) |
| Substantia Nigra | 1.18 (±0.19) | 1.31 (±0.03) |

All *P*> 0.05, Bonferroni corrected for multiple comparisons.

## Table S4 [^18^F]AV1451 Logan DVR and SUVR values in anatomical brain regions in the group of healthy controls.

| Regions of Interest | Logan DVR (mean±SD) | SUVR (mean±SD) |
| --- | --- | --- |
| Frontal Lobe | | |
| Superior Frontal gyrus | 0.93 (±0.09) | 1.03 (±0.11) |
| Middle Frontal gyrus | 0.95 (±0.10) | 1.07 (±0.10) |
| Inferior Frontal gyrus | 0.97 (±0.10) | 1.08 (±0.11) |
| Precentral gyrus | 0.90 (±0.09) | 1.02 (±0.11) |
| Parietal Lobe | | |
| Angular gyrus | 0.97 (±0.11) | 1.07 (±0.11) |
| Superior Parietal gyrus | 0.95 (±0.10) | 1.04 (±0.11) |
| Postcentral gyrus | 0.88 (±0.09) | 0.98 (±0.09) |
| Supramarginal gyrus | 0.93 (±0.09) | 1.04 (±0.09) |
| Occipital Lobe | | |
| Lateral Occipital Lobe | 0.95 (±0.10) | 1.05 (±0.08) |
| Lingual gyrus | 0.98 (±0.10) | 1.04 (±0.11) |
| Cuneus | 0.96 (±0.12) | 1.03 (±0.10) |
| Temporal Lobe | | |
| Posterior Temporal Lobe | 0.99 (±0.09) | 1.11 (±0.07) |
| Superior Temporal gyrus | 0.96 (±0.10) | 1.04 (±0.06) |
| Middle and Inferior Temporal gyrus | 1.03 (±0.10) | 1.16 (±0.08) |
| Fusiform gyrus | 0.97 (±0.07) | 1.10 (±0.10) |
| Anterior Cingulate | 0.92 (±0.10) | 1.00 (±0.09) |
| Posterior Cingulate | 1.02 (±0.10) | 1.08 (±0.09) |
| Subcortical brain regions | | |
| Amygdala | 0.97 (±0.11) | 1.11 (±0.09) |
| Brainstem | 0.87 (±0.11) | 0.98 (±0.10) |
| Caudate | 0.91 (±0.11) | 1.01 (±0.08) |
| Putamen | 1.29 (±0.14) | 1.45 (±0.17) |
| Globus Pallidus | 1.37 (±0.17) | 1.59 (±0.27) |
| Thalamus | 1.11 (±0.11) | 1.24 (±0.14) |
| Nucleus Accumbens | 1.12 (±0.12) | 1.26 (±0.13) |
| Substantia Nigra | 1.17 (±0.19) | 1.39 (±0.03) |

All *P*> 0.05, Bonferroni corrected for multiple comparisons.

## Table S5 Voxel-based analysis of [^18^F]AV1451 in patients with Corticobasal Syndrome, Mild Cognitive Impairment and healthy controls.

| MNI coordinates | | | Area | Cluster Size | | Z-scores | *P* value* |
| --- | --- | --- | --- | --- | --- | --- | --- |
| X | **Y** | **Z** |  |  |  |  |  |
| Increased [^18^F]AV1451 in CBS compared to HC | | | | | | | |
| -26 | 16 | 48 | Middle Frontal Gyrus L | | 43781 | 4.22 | <0.001 |
| -24 | 4 | 58 | Superior Frontal Gyrus L | | 43781 | 3.28 | 0.001 |
| -26 | 16 | 48 | Dorsolateral Frontal L | | 27543 | 4.22 | <0.001 |
| 42 | -6 | 38 | Precentral Gyrus R | | 1324 | 3.29 | <0.001 |
| -50 | 6 | 34 | Precentral Gyrus L | | 27543 | 3.55 | <0.001 |
| -14 | 20 | 52 | Posterior Medial Frontal L | | 43781 | 3.48 | <0.001 |
| -36 | 32 | 10 | Anterior Dorsolateral Frontal L | | 415 | 1.83 | 0.034 |
| Increased [^18^F]AV1451 in CBS compared to MCI | | | | | | | |
| 34 | -1 | 57 | Posterior Dorsolateral Frontal R | | 18349 | 3.15 | <0.001 |
| -24 | -79 | 35 | Posterior Dorsolateral Frontal L | | 18349 | 2.97 | <0.001 |
| 12 | -53 | 63 | Parietal Lobe R | | 18349 | 3.1 | <0.001 |
| -52 | -43 | 21 | Supramarginal Gyrus L | | 21 | 1.82 | 0.035 |
| Increased [^18^F]AV1451 in MCI compared to CBS | | | | | | | |
| 60 | -35 | -23 | Inferior Temporal Gyrus R | 1173 | | 2.97 | <0.001 |
| -64 | -35 | -19 | Middle Temporal Gyrus L | 1173 | | 1.97 | 0.025 |
| -52 | -43 | 19 | Superior Temporal Gyrus L | 282 | | 1.82 | 0.034 |
| -42 | -59 | -21 | Fusiform Gyrus | 110 | | 1.87 | 0.031 |

**P* values are FWE corrected. CBS=Corticobasal Syndrome, HC=healthy controls; MCI=Mild Cognitive Impairment.

## Table S6 [^18^F]AV45 SUVR in anatomical brain regions in the group of Corticobasal Syndrome and Mild Cognitive Impairment patients.

| Regions of Interest | CBS  (mean±SD) | MCI (mean±SD) | *P* value* |
| --- | --- | --- | --- |
| Hippocampus | 1.14 (±0.18) | 1.30 (±0.16) | **0.015** |
| Amygdala | 1.00 (±0.13) | 1.21 (±0.22) | **0.004** |
| Parahippocampal gyrus | 1.02 (±0.11) | 1.18 (±0.17) | **0.008** |
| Superior Frontal gyrus | 1.08 (±0.12) | 1.32 (±0.24) | **0.014** |
| Middle Frontal gyrus | 1.12 (±0.11) | 1.46 (±0.25) | **<0.001** |
| Precentral gyrus | 1.05 (±0.08) | 1.42 (±0.26) | **<0.001** |
| Postcentral gyrus | 1.02 (±0.07) | 1.37 (±0.25) | **<0.001** |
| Angular gyrus | 1.14 (±0.18) | 1.35 (±0.26) | **0.01** |
| Superior Parietal gyrus | 1.10 (±0.12) | 1.35 (±0.26) | **<0.001** |

All *P* values are Bonferroni corrected for multiple comparisons. CBS=Corticobasal Syndrome; MCI=Mild Cognitive Impairment.

## Table S7 Volumetric brain changes in patients with Corticobasal Syndrome, Mild Cognitive Impairment and healthy controls.

| Regions of Interest | HC  (mean±SD) | CBS  (mean±SD) | MCI  (mean±SD) | *CBS vs HC*  *P* value | *CBS vs MCI*  *P* value |
| --- | --- | --- | --- | --- | --- |
| Superior Frontal gyrus | 2.46 (±0.15) | 2.43 (±0.22) | 2.45 (±0.17) | >0.10 | >0.10 |
| Middle Frontal gyrus | 2.24 (±0.17) | 2.06 (±0.14) | 2.24 (±0.17) | **0.007** | **0.006** |
| Precentral gyrus | 2.32 (±0.22) | 2.13 (±0.17) | 2.32 (±0.20) | **<0.001** | **0.009** |
| Postcentral gyrus | 1.92 (±0.16) | 1.84 (±0.15) | 1.92 (±0.14) | >0.10 | >0.10 |
| Supramarginal gyrus | 2.36 (±0.14) | 2.15 (±0.18) | 2.36 (±0.15) | **0.008** | **0.006** |
| Superior Parietal gyrus | 2.03 (±0.18) | 1.98 (±0.18) | 2.04 (±0.14) | >0.10 | <0.001 |
| Enthorinal cortex | 2.81 (±0.57) | 3.04(±0.68) | 2.38 (±0.57) | >0.10 | **0.016** |
| Temporal Pole | 2.95 (±0.80) | 3.34 (±0.13) | 2.62 (±0.22) | >0.10 | **0.007** |
| Caudate | 2.39 (±0.34) | 2.27 (±0.70) | 2.25 (±0.24) | >0.10 | >0.10 |
| Putamen | 2.87 (±0.40) | 2.74 (±0.73) | 2.88(±0.43) | >0.10 | >0.10 |
| Globus Pallidus | 1.03 (±0.20) | 0.90 (±0.22) | 0.94 (±0.16) | >0.10 | >0.10 |

All *P* values are Bonferroni corrected for multiple comparisons. CBS=Corticobasal Syndrome; HC=healthy controls; MCI=Mild Cognitive Impairment.

## Table S8 Microstructural white matter changes in the group of Corticobasal Syndrome compared to healthy controls.

| Regions of Interest | HC  (mean±SD) | CBS  (mean±SD) | *MCI* | *CBS vs HC*  *P* value | *CBS vs MCI*  *P* value |
| --- | --- | --- | --- | --- | --- |
| *FA* | | | | | |
| Superior Frontal gyrus | 0.219 (±0.01) | 0.203 (±0.02) | 0.210 (±0.07) | **0.039** | >0.10 |
| Middle Frontal gyrus | 0.231 (±0.02) | 0.215 (±0.02) | 0.220 (±0.02) | >0.10 | >0.10 |
| Precentral gyrus | 0.264 (±0.02) | 0.243 (±0.05) | 0.249 (±0.08) | **0.037** | >0.10 |
| Postcentral gyrus | 0.245 (±0.01) | 0.237 (±0.02) | 0.233 (±0.01) | >0.10 | >0.10 |
| Angular gyrus | 0.186 (±0.02) | 0.164 (±0.01) | 0.177 (±0.06) | **0.008** | >0.10 |
| Superior Parietal gyrus | 0.234 (±0.02) | 0.214 (±0.01) | 0.223 (±0.07) | **0.035** | >0.10 |
| *MD* | | | | | |
| Superior Frontal gyrus | 0.0011 (±0.0) | 0.0010 (±0.0) | 0.0011 (±0.0) | >0.10 | >0.10 |
| Middle Frontal gyrus | 0.0011 (±0.0) | 0.0010 (±0.0) | 0.0010 (±0.0) | **0.013** | >0.10 |
| Precentral gyrus | 0.0011 (±0.0) | 0.0010 (±0.0) | 0.0010 (±0.0) | **0.018** | **0.042** |
| Postcentral gyrus | 0.0012 (±0.0) | 0.0010 (±0.0) | 0.0010 (±0.0) | **0.001** | **0.020** |
| Angular gyrus | 0.0012 (±0.0) | 0.0010 (±0.0) | 0.0010 (±0.0) | **0.007** | >0.10 |
| Superior Parietal gyrus | 0.0012 (±0.0) | 0.0010 (±0.0) | 0.0011 (±0.0) | **0.001** | **0.034** |
| Supramarginal gyrus | 0.0010 (±0.0) | 0.0013 (±0.0) | 0.0010 (±0.0) | **0.008** | **0.002** |

All *P* values are Bonferroni corrected for multiple comparisons. CBS=Corticobasal Syndrome; FA=fractional anisotropy; HC=healthy controls; MCI=Mild Cognitive Impairment; MD=mean diffusivity.

## Figure S1 [^18^F]AV1451 Logan DVR and SUVR_60-80_ values in anatomical brain regions in the group of healthy controls and Corticobasal Syndrome patients.

**
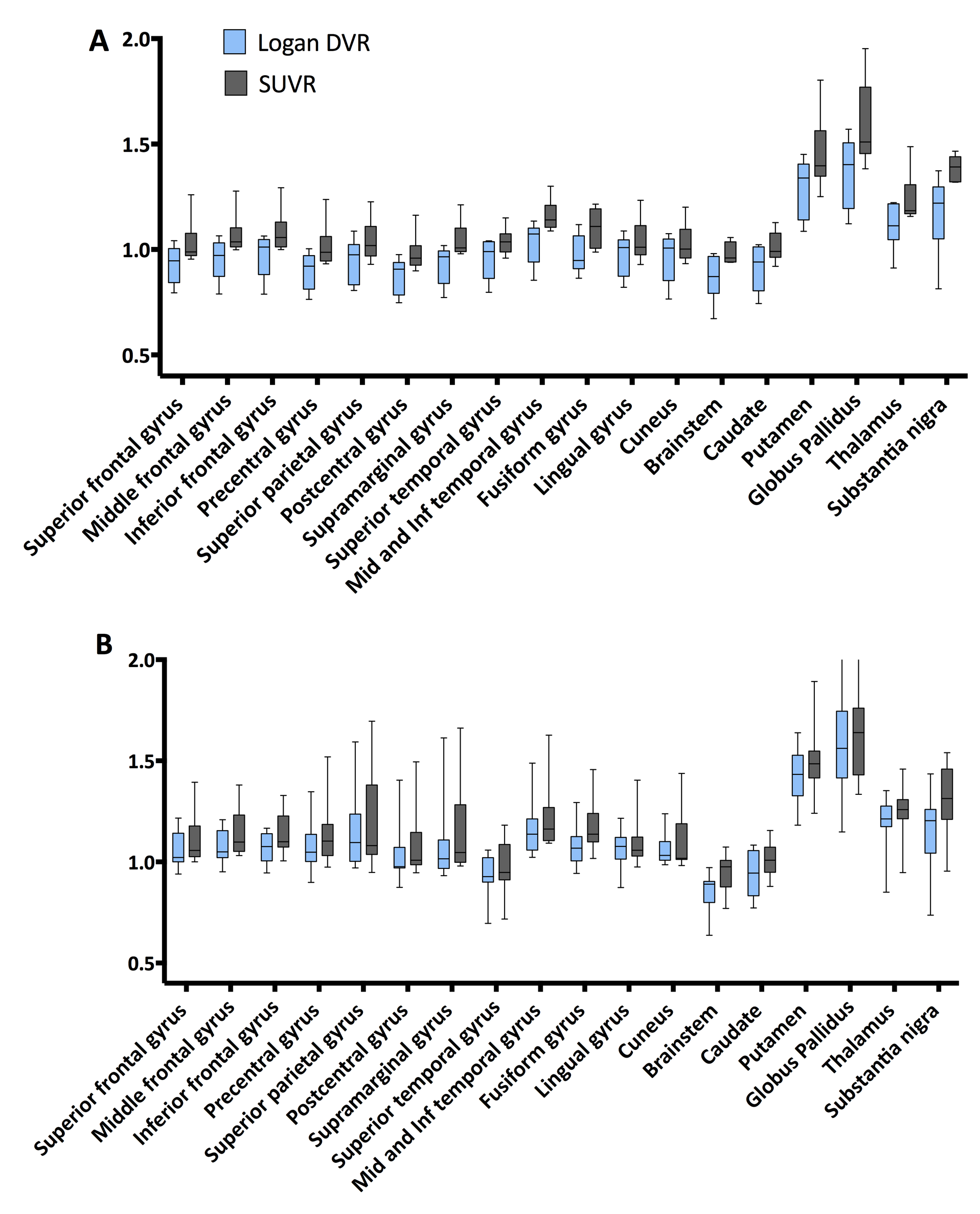
**

Bar graph showing cortical and subcortical [^18^F]AV1451 Logan DVR ***(***V_T_^tissue^ /V_T_^ref^) and SUVR in patients with CBS (A) and healthy controls (B). Whiskers indicate variability outside the upper and lower quartiles, the median is marked by a horizontal line inside the box. **P*<0.05. All *P* values are Benjamini-Hochberg corrected for multiple comparisons.

## Figure S2 Increased Tau deposition in anatomically defined brain regions of Corticobasal Syndrome patients.


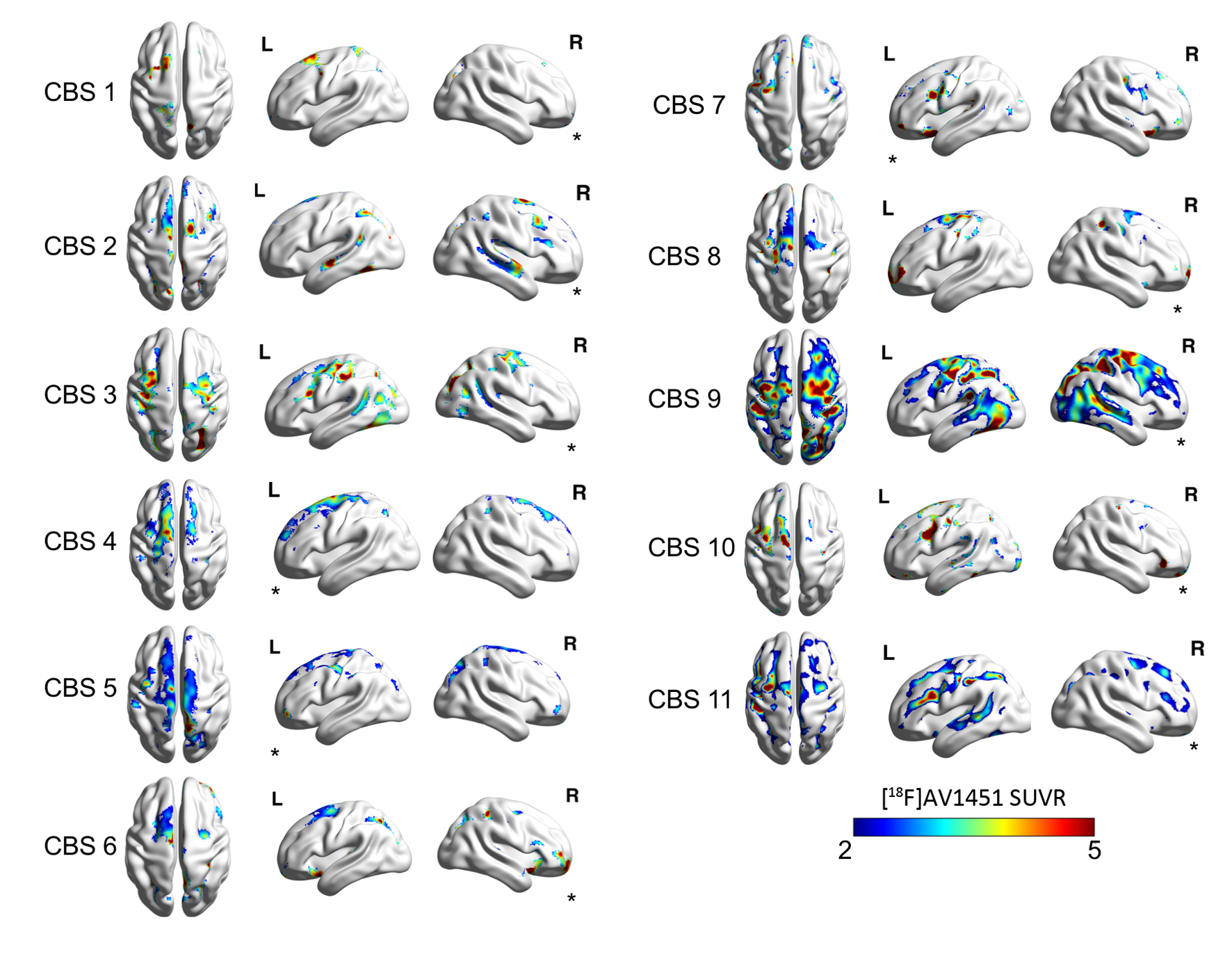


Voxel-wise z-score maps for [^18^F]AV1451 standardized uptake value ratios (SUVR) for each CBS patient against all healthy controls. *The most affected side of the brain in each patient with CBS.

## Figure S3 Tau and Amyloid-β deposition in anatomically defined brain regions of Corticobasal Syndrome patients.


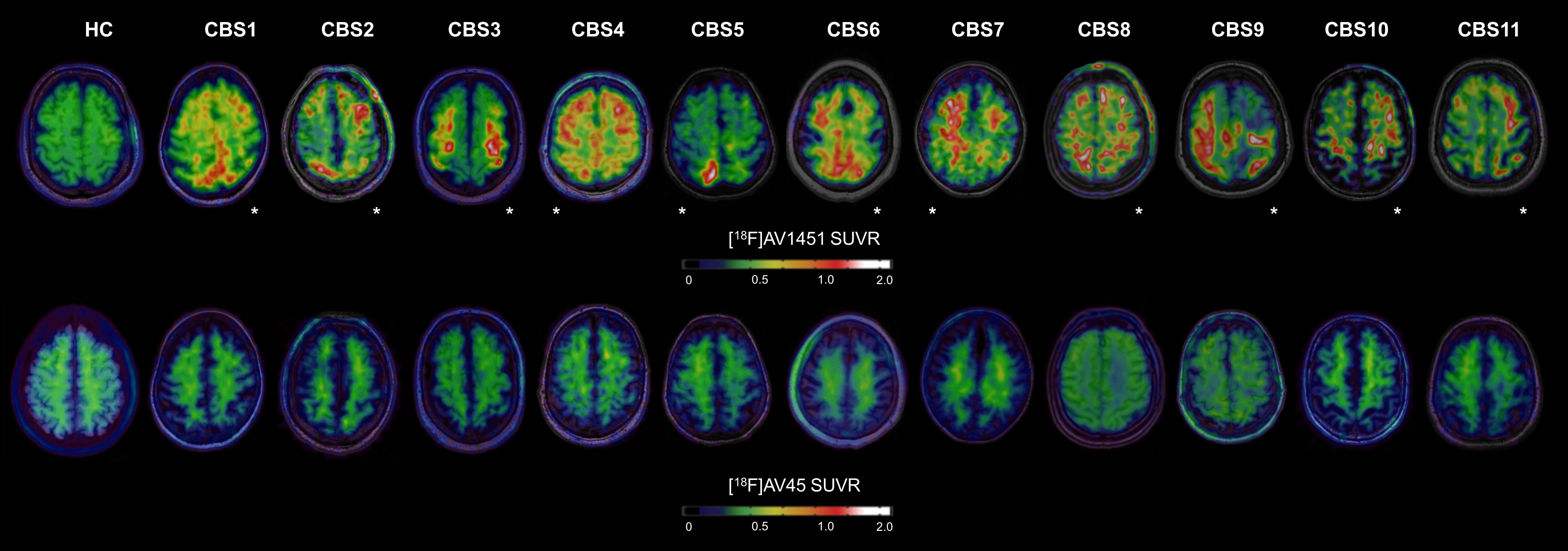


Axial summed [^18^F]AV1451 and [^18^F]AV45 PET images fused co-registered and fused with 3T MRI images for the cortex of a healthy control and 11 CBS patients. CBS=Corticobasal Syndrome. Colour bar reflects range of [^18^F]AV1451 SUVR and [^18^F]AV45 SUVR intensity. *Contralateral to the clinically most affected body side in each patient (the images are in neurologic orientation).

## Figure S4 Statistical parametric maps of [^18^F]AV1451 SUVR in groups of patients with CBS and MCI compared to healthy controls.


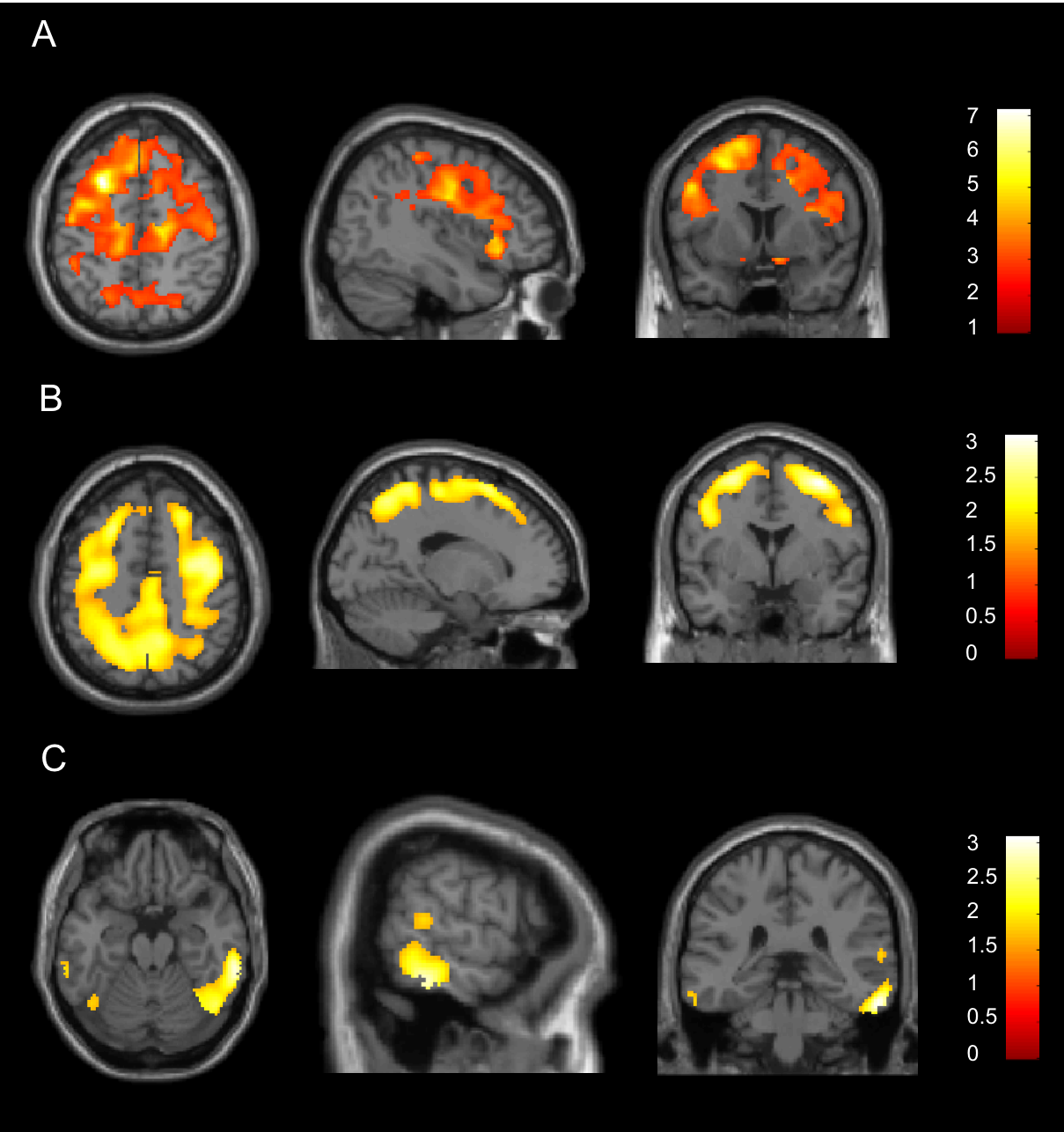


Yellow-red areas represent voxel clusters with significant increases in [^18^F]AV1451 uptake in (A) patients with CBS compared to healthy controls, (B) patients with CBS compared to MCI and (C) patients with MCI compared to CBS patients. The colour stripe indicates z values. MNI co-ordinates X=41; Y=2; Z=46. CBS=Corticobasal Syndrome; HC=Healthy Controls; MCI=Mild Cognitive Impairment.

## Figure S5 Correlations between gray matter and white matter changes and clinical measures in the cohort of Corticobasal Syndrome patients.

##
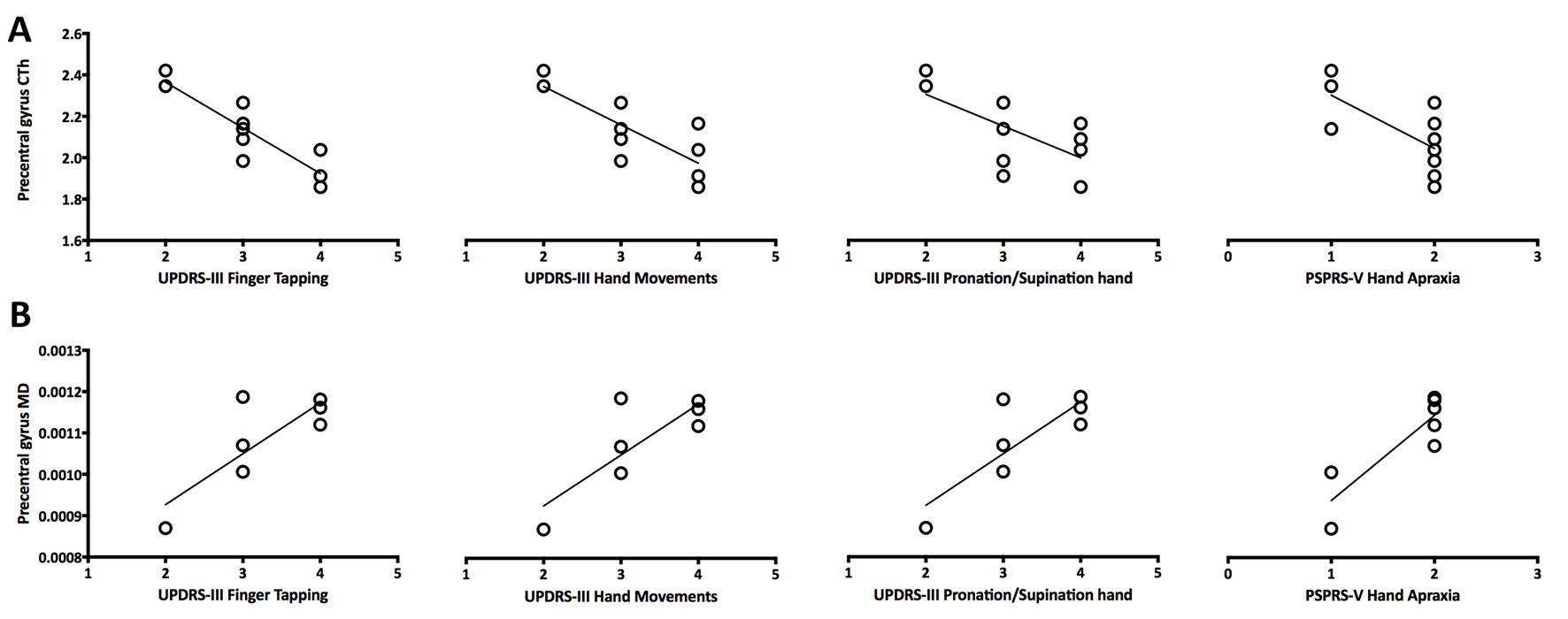


Scatterplots showing correlations between **A)** Decreased cortical thickness in the precentral gyrus contralateral to the clinically most affected body side and worse motor scores at the finger tapping (UPDRS-III Item 3.4; *r_s_*=-0.86; *P*=0.001), hand movements (UPDRS-III Item 3.5; *r_s_*=-0.78; *P*=0.008), pronation/supination movements of the hand (UPDRS-III Item 3.6; *r_s_*=-0.71; *P*=0.022) and apraxia of hand movement (PSPRS Item 22; *r_s_*=-0.68; *P*=0.031) of the clinically most affected body side; **B)** Increased MD values in the precentral gyrus contralateral to the clinically most affected body side and worse motor scores at the finger tapping (UPDRS-III Item 3.4; *r_s_*=0.81; *P*=0.027), hand movements (UPDRS-III Item 3.5; *r_s_*=0.81; *P*=0.027), pronation/supination movements of the hand (UPDRS-III Item 3.6; *r_s_*=0.82; *P*=0.024) and apraxia of hand movement (PSPRS Item 22; *r_s_*=0.87; *P*=0.010) of the clinically most affected body side.

## Figure S6 Correlation between FA values and clinical measures in the cohort of Corticobasal Syndrome patients.


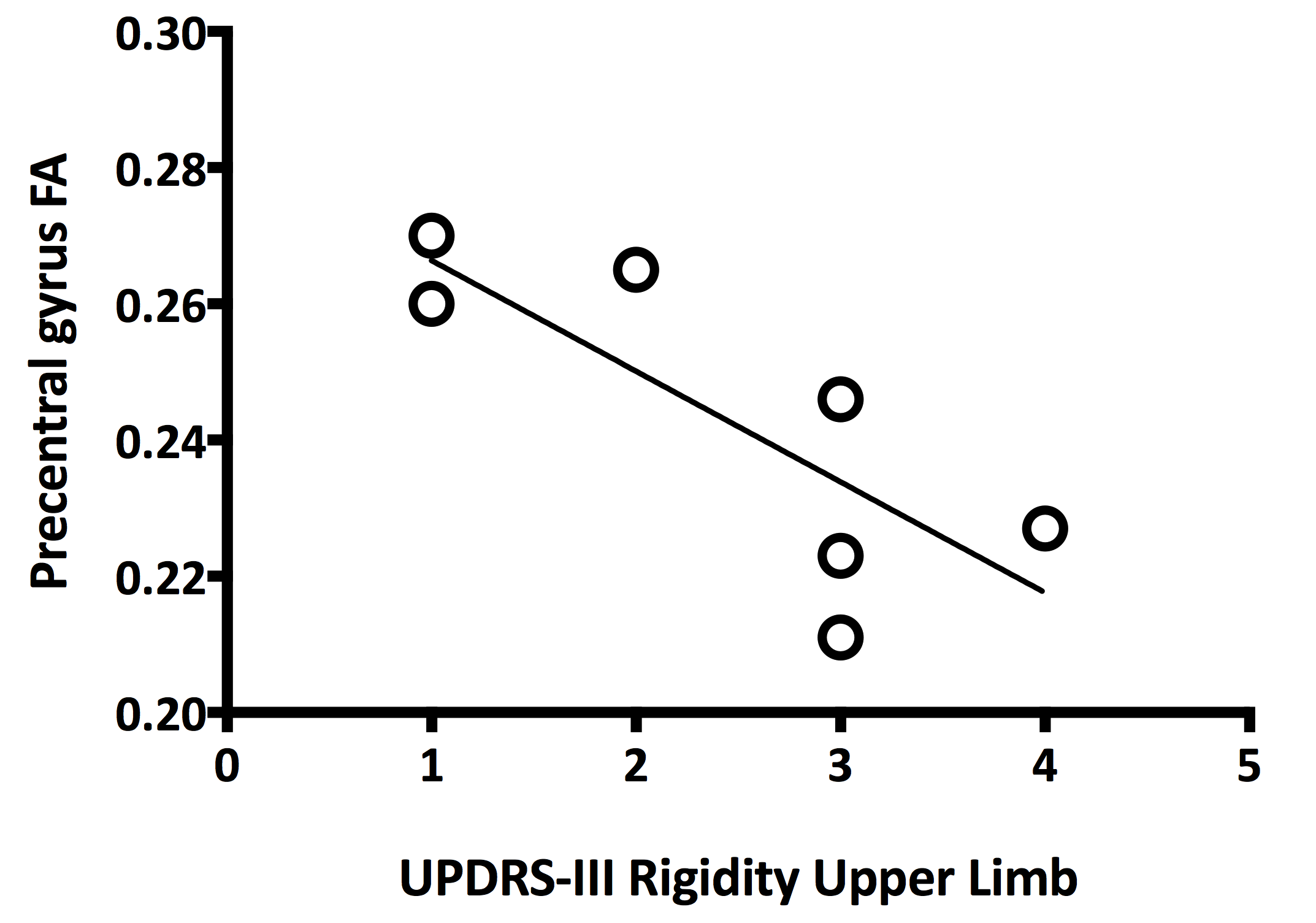


Scatterplot showing significant correlation between decreased FA values in the precentral gyrus contralateral to the clinically most affected body side and upper limb rigidity (UPDRS-III Item 3.3) in the clinically most affected side (*r_s_*=-0.80; *P*=0.031).

## Figure S7 Correlation between [^18^F]AV1451 SUVR and clinical measures in the cohort of Corticobasal Syndrome patients.


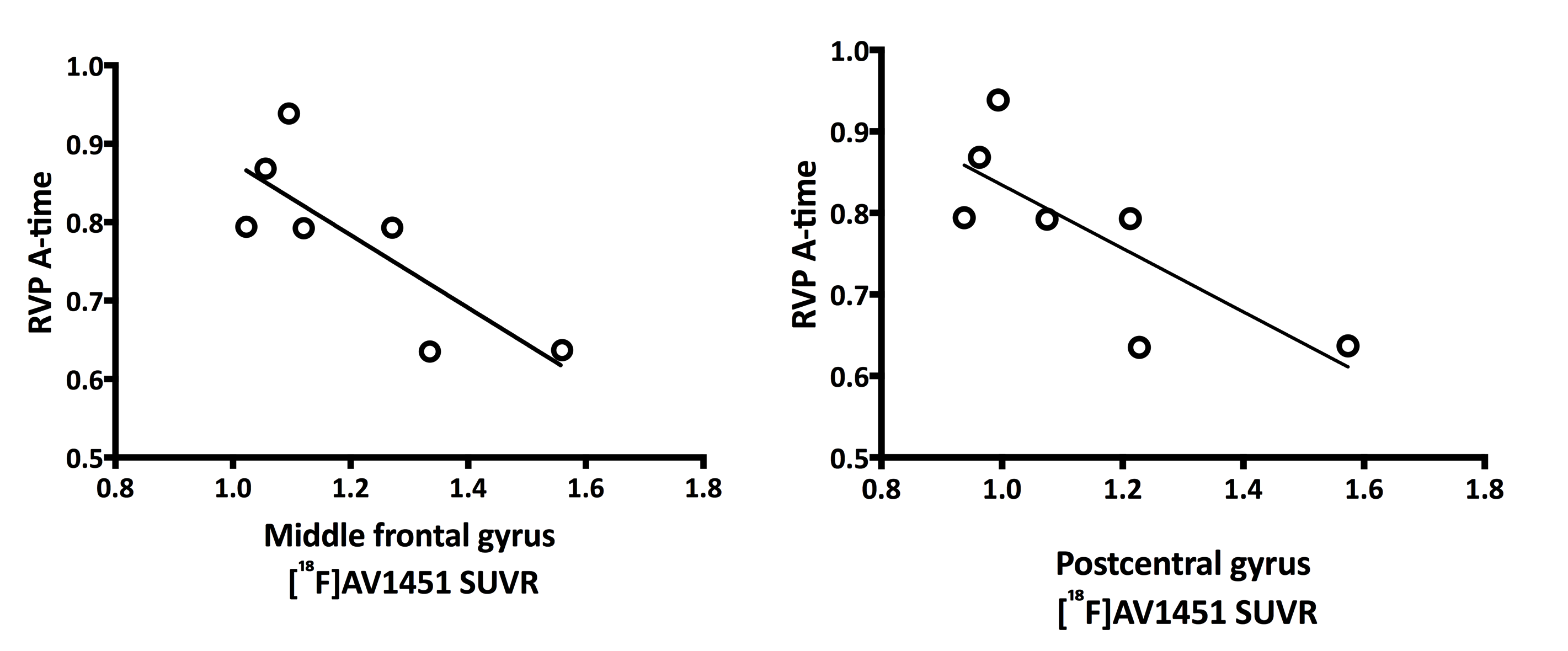


Scatterplots showing significant correlation between decreased Rapid Visual Information Processing (RVP) A-time scores and increased [^18^F]AV1451 SUVR in middle frontal gyrus (*r_s_*=-0.79; *P*=0.036) and postcentral gyrus (*r_s_*=-0.79; *P*=0.036) contralateral to the clinically most affected body side.

# REFERENCES

1. Jagust WJ, Bandy D, Chen K et al. The Alzheimer’s disease neuroimaging initiative positron emis- sion tomography core. Alzheimers Dement 2010; 6: 221–9.
2. Heckemann RA, Keihaninejad S, Aljabar P et al. Alzheimer’s Disease Neuroimaging Initiative. Improving intersubject image registration using tissue-class information benefits robustness and accuracy of multi-atlas based anatomical segmentation. Neuroimage 2010;51:221–227.
3. Friston KJ, Holmes AP, Worsley KJ et al. Statistical parametric maps in functional imaging: a general linear approach. Hum Brain Mapp 1995; 2:189–210.
